# Environmental modulation of methyl farnesoate release in two clones of *Daphnia pulex* differing in the sensitivity to exogenous stimuli

**DOI:** 10.1371/journal.pone.0361189

**Published:** 2026-10-27

**Authors:** Nicolo’ Bellin, Nicolo’ Riboni, Annamaria Buschini, Federica Bianchi, Maria Careri, Chiara Conciatori, Clarissa Facchini, Laura Filonzi, Stefano Nicolosi, Valeria Rossi

**Affiliations:** 1 Department of Chemistry, Life Sciences and Environmental Sustainability, University of Parma, Parma, Italy; 2 Department of Biology, University of Puerto Rico, San Juan, Puerto Rico; Universidad Nacional Autonoma de Mexico, MEXICO

## Abstract

*Daphnia* is a widely recognised model organism in ecotoxicological studies, exhibiting environmental sex determination. Environmental cues such as temperature, photoperiod and crowding are known to trigger transitions from parthenogenetic to sexual reproduction. Experimental studies based on exogenous exposure suggest that methyl farnesoate (MF), a juvenile hormone–like compound, is associated with male production and predator-induced defensive traits in *Daphnia*; however, direct evidence of endogenous MF production and environmental dynamics remains limited. In this study, we investigated the release of endogenous MF into the rearing water of *Daphnia pulex.* Two *D. pulex* clonal lineages differing in propensity for male, ephippia and neckteeth production were selected. Experimental conditions simulating crowding and different habitat suitability, including variations in population density, food availability, photoperiod and temperature, were investigated. MF was quantified at ultra-trace levels using solid-phase microextraction coupled to gas chromatography–tandem mass spectrometry. In the first generation, MF concentrations decreased from approximately 18.5–20.0 ng/L in controls to 11.0–14.5 ng/L under crowding conditions and 6.7–7.2 ng/L under crowding combined with MK-801. In the second generation, concentrations were approximately 10.5 ng/L in control offspring and 12.0–12.3 ng/L after recovery from crowding. Exposure to crowding over two generations resulted in clone-specific concentrations, whereas MF remained below the limit of quantification of 0.08 ng/L, when parental females had been exposed to MK-801. In the density and seasonal experiments, MF released in the medium was higher release per female under early-spring conditions and in clone S. These variations occurred under conditions promoting male production, ephippia formation and antipredator responses. While not demonstrating a causal endocrine role of MF, our findings provide quantitative and environmentally relevant evidence that *D. pulex* releases MF into the aquatic environment and this release is modulated by both environmental conditions and genetic background.

## Introduction

*Daphnia* is a well-established model organism in ecotoxicology and is routinely employed in standardized tests for the assessment of chemical substances and environmental samples. Its high sensitivity to pollutants and environmental stressors makes it a key indicator species for freshwater ecosystem health [1]. Several environmental contaminants have been shown to disrupt endocrine-related pathways in *Daphnia*; in particular, compounds with juvenile hormone (JH)-like activity are known to interfere with crucial life-history traits, including reproduction and development [2]. In this context, methyl farnesoate (MF) has been proposed as a potential molecular target of endocrine-disrupting compounds, given its structural and functional similarity to insect juvenile hormones [2].

MF is the unoxidized precursor of juvenile hormone III (JH III) in insects and has been detected in more than 30 species of crustaceans. Juvenile hormones and JH‑like compounds are known to regulate a wide range of developmental and physiological processes in arthropods, including moulting, growth, reproduction, polyphenism, cyclomorphosis, and dormancy [3–9]. In cladocerans, experimental evidence based on exposure to exogenous JH analogues or MF suggests an association between elevated JH-like signalling and the induction of male offspring as well as predator-induced defensive morphologies [10–13]. For example, exposure to juvenile hormone III induced male production with an EC50 of approximately 4 µM, whereas MF concentrations of 48–50 µg/L produced an approximately 1:1 offspring sex ratio in *D. magna* [7]. Methoprene concentrations up to 160 nM did not induce male production under female-promoting conditions but produced a concentration-dependent response when combined with crowding and food restriction [7]. These findings demonstrate that JH-like compounds can alter sex determination; however, the concentrations used in exogenous exposure experiments are substantially higher than the endogenous MF levels expected in the surrounding aquatic medium. However, the extent to which MF acts as an endogenous juvenile hormone in *Daphnia*, and whether it directly mediates these processes under natural conditions, remains a matter of debate.

In *D. pulex*, MF has been proposed as a candidate endogenous JH‑like compound based on genetic and functional studies indicating modifications in the JH III biosynthetic pathway [14,15]. However, available evidence mainly derives from phenotypic responses to exogenous MF, pharmacological manipulation of the putative signaling pathway, or changes in the expression of genes involved in MF/JH biosynthesis. MF has not yet been chemically characterized within *Daphnia* tissues or hemolymph. The detection of MF in *Daphnia* culture water provides indirect evidence of its release but does not establish its synthesis within the organism or demonstrate that environmentally induced variations in endogenous MF directly cause reproductive or defensive phenotypes. As highlighted by Miyakawa, although MF is considered a central to the regulation of reproductive strategy shifts and phenotypic plasticity in response to environmental cues, the endogenous occurrence and chemical identity of the active JH-like compound in *Daphnia* remain unresolved [13].

*Daphnia* exhibits environmental sex determination, with no evidence for sex chromosomes [16]. Under favourable environmental conditions, populations are dominated by parthenogenetically reproducing females, enabling rapid clonal population growth. In cyclic parthenogenetic populations, the production of males and ephippia (resistant resting eggs containing diapausing embryos) is triggered by environmental cues such as changes in photoperiod, temperature, food quality and availability, crowding, and predator-associated chemical signals [17–21]. These transitions are generally assumed to involve endocrine regulation, although the identity and environmental dynamics of the endogenous mediators involved remain incompletely resolved. Predator-induced morphological plasticity, such as neckteeth formation in juvenile *D. pulex*, represents an additional defensive response linked to environmental stress [22–24] and has been shown to overlap, at the molecular level, with pathways associated with male production following exposure to exogenous MF or JH analogues [8,12,25,26]. A seasonal fluctuation in the frequency of different *D. pulex* morphotypes depending on seasonal variations of photoperiod, temperature, food availability, and population density, which could act as a signal of predation risk was also described [27–29].

Pharmacological experiments indicate that inhibition of upstream signalling pathways, such as those involving the N‑methyl‑D‑aspartate (NMDA) receptor, suppresses male production in specific *D. pulex* strains—an effect reversible by the administration of exogenous MF [7,9,10,30]. These results support the hypothesis that MF may participate in stress‑related developmental pathways, although they do not provide direct evidence for its endogenous synthesis, release, or mode of action under natural conditions.

The analytical detection of MF at ultra-trace levels is challenging and requires highly sensitive and selective methodologies. MF has been detected in tissues and organs of crustaceans and insects using nuclear magnetic resonance [31], liquid chromatography coupled to UV‑vis detection [32], or mass spectrometry [33]. However, gas chromatography coupled to mass spectrometry (GC–MS) remains the most widely applied approach due to its sensitivity and selectivity. Riboni et al. reported the detection of MF at ultra‑trace levels in water from cultures of *D. pulex* reared without exogenous MF administration (up to 20 ng/L), using solid‑phase microextraction (SPME) coupled to GC–MS [34]. This result provided indirect but compelling evidence that *Daphnia* may release MF into the surrounding aquatic environment.

In the present study, we investigate the relationship between life-history traits and the release of MF into the rearing water by two clones (I and S) of *D. pulex* differing in their propensity for male, ephippia, and neckteeth production [18,35]. We hypothesise that environmental conditions known to induce reproductive and defensive responses may be associated with measurable changes in the amount of MF released into the surrounding water, consistent with its potential involvement in stress‑related developmental pathways. Two experiments were conducted: the first assessed MF release over two successive generations in response to crowding conditions and following exposure to MK‑801, an NMDA receptor antagonist known to interfere with JH‑like signalling [9,10]; the second evaluated MF release under environmental conditions simulating early and late spring, differing in photoperiod and temperature. MF quantification was performed using gas chromatography–tandem mass spectrometry (GC–MS/MS) to improve both method sensitivity and selectivity. Finally, MF release patterns were analysed in relation to male, ephippia, and neckteeth production.

## Materials and methods

### SPME-GC-MS/MS analysis

SPME extraction was carried out according to the procedure previously optimized by Riboni et al. [34]. Briefly, 9.5 mL of water was extracted using a 1 cm–50/30 μm DVB-CAR-PDMS fiber by operating in direct immersion mode under an agitation speed of 250 rpm at 80 °C for 50 min. After extraction the fiber was desorbed into the GC injection port at 260°C. GC-MS/MS analyses were performed using a GC-2030 Nexis gas chromatograph coupled with a TQ8040 NX mass spectrometer (Shimadzu, Kyoto, Japan), equipped with a RTX-5 MS capillary column (30 m x 0.25 mm i.d., 0.25 μm film thickness, Restek, Bellafonte, KY, USA) and an AOC-6000 Plus autosampler (Shimadzu, Kyoto, Japan). Helium was used as carrier gas at a constant flow rate of 1.2 mL/min and the following oven temperature program was used: 90°C hold for 2 min, 15°C/min to 180°C, 20°C/min to 270°C. Transfer line and ion source were maintained at 280 and 260°C, respectively; an electron ionization energy value of 70 eV and an electron multiplier voltage of 1.55 kV were set. A multiple reaction monitoring (MRM) mode was used for tandem MS/MS analysis. The precursor ions were fragmented by collision-induced dissociation (CID) using argon and collision energies in the 5–25 eV range. The molecular ions of both MF (m/z 250) and methyl decanoate (internal standard-IS, m/z 186) were selected as precursor ions. The following transitions (in bold those used for quantitation) were monitored: 250 **→** 69, 250 **→** 114, 250 → 121 and for MF and 186 → 74, 186 → 87 and 186 → 121 for the IS (S7 Fig). Method validation was performed according to EURACHEM guidelines, using bottled water and food as blank matrix (S1 Table) [36]. Blank matrix was exposed to the same environmental conditions applied during the experiments with *Daphnia*.

### *D. pulex* culturing

In the laboratory, females were singly bred in a commercial natural water (NW; San Benedetto, Scorzè, Italy) at 20 ± 1 °C with photoperiod of 14:10 (L:D) h, fed with the unicellular green alga *Pseudokirchneriella subcapitata* and the yeast *Saccharomyces cerevisiae* at a density of 1.5x10^5^ cells/mL, twice a week and maintained at a population density of 20 females per clone in 600 ml of the breading water that was renewed twice a week.

### Experiment 1: Effects of crowding due to a reduction of water volume and addition of MK-801

MK-801 [(5S,10R)-(+)-5-Methyl-10,11-dihydro-5H-dibenzo[a,d]cycloheptenen-5,10-imine maleate] (    ≥ 98% purity) was from Merck, Milan, Italy.

In the G1 life-table experiment, female juveniles (less than 24-h old, produced in the third clutch), were isolated from the clonal culture(s) and randomly assigned to each treatment: control (Contr,1 individual/50 mL), crowding (Crow, 1 individual/10 mL), and crowding + MK-801 (MK, individual/10 mL + 1μM MK-801).

In the G2 life-table experiment, G2 female juveniles of each clone (less than 24-h old, produced in the third clutch of G1 females) were exposed to the six different treatments: 1) ContrContr: G2 control daphnids born to G1 control females; 2) CrowContr: G2 control daphnids born to G1 females exposed to crowding; 3) CrowCrow: both generations (G1 and G2) exposed to crowding; 4) MKContr: G2 control daphnids born to G1 females exposed to MK-801; 5) MKCrow: G2 daphnids exposed to crowding born to G1 females exposed to MK-801; 6) MKMK: both generations (G1 and G2) exposed to MK-801.

The water samples were stored at −20°C to minimize MF losses due to evaporation and to prevent potential chemical or enzyme-mediated degradation before SPME-GC-MS/MS analysis. Body size (as carapace length from the top of the head to the base of the tail spine) was measured on the 8^th^ day of treatment); age at maturity (the age at which the first eggs are deposited into the brood chamber) and fecundity (total number of alive offspring per female) were recorded three times a week. Juveniles were removed and classified according to their gender using a CLS 50x microscope (Leica CLS 50x microscope; Leica, Mannheim, Germany).

### Experiment 2: Effects of crowding due to increasing density and seasonal conditions

To confirm the release of MF by *D. pulex* and to assess whether the concentration of endogenous MF released was related to males, ephippia and neckteeth production, clones I and S were reared at 5 different densities and 2 different experimental conditions for one generation (see Supplementary information).

Water samples were collected during renewal at densities 1, 2 and 5 females per beaker after 8, 14 and 21 days, allowing MF released by adult females to accumulate in the rearing medium over 48 h. The water samples were stored at −20°C and then analysed by SPME-GC-MS/MS.

### Statistical analysis

As for the statistical analysis on the biological assays*,* in the first experiment, for both G1 and G2, a Bayesian framework for Generalized Linear Models (GLM) based on Markov Chain Monte Carlo sampling [37]. was used to estimate the posterior distribution of life table variables. The carapace length (mm) was estimated considering a gaussian family model and a normal prior for the intercept parameter (mean = 1.91 mm, sd = 0.12 mm) was used from previous experimental measurement (unpublished data). Neonates per female and age at first deposition (days) were estimated using a Poisson model, while the male’s production was fitted with a Poisson zero inflated family function. For each Bayesian model the predictive factors were clone, type of treatment (G1: Contr, Crow, MK and G2: ContrContr, CrowContr, CrowCrow, MKContr, MKCrow and MKMK) and their interaction. For each model, credibility intervals (2.5 and 97.5% C.I.) were computed according to the posterior distribution of the life table variables and used to compare each experimental treatment.

In the second experiment, the same model families of the first experiment were used to estimate the carapace size (mm) and neonates per female. For both life table variables, prior distribution of the gaussian family was estimated from the first experiment and set into the model (carapace length: mean = 2.07, sd = 0.18 and neonates per female: mean = 5.52, sd = 0.17). For each Bayesian GLM model, clone, density, environmental conditions and their interactions were used as predictive factors. All models were fitted with brms R package [38] using 4 chains, with 10000 iterations and a warmup phase of 2000.

In the second experiment, the production of males and ephippia in both environmental conditions and densities were recorded. Differences in age at first deposition between environmental conditions and clones were tested with the Brown-Mood median test using the R package coin [39].

The χ^2^ test was performed to assess differences in the frequency of the different morphotypes between treatments, between clones and densities. Differences were considered significant at *p*-values lower than the 0.05 threshold. The χ^2^ test was performed using the R function chisq.test of the stats package.

The relationships between the concentration of MF released in the water with male and ephippia production were modelled using a nonlinear least-squares estimates of an exponential decay model, while the percentage of morphotypes among clones was fit with a linear regression model using a Bayesian estimate with 4 chains, with 4000 iterations and a warmup phase of 2000.

The data and R scripts used for the statistical analyses are available in Zenodo at https://doi.org/10.5281/zenodo.22935162.

## Results

### SPME-GC-MS/MS method validation

The use of a triple quadrupole tandem mass spectrometer operating in MRM mode resulted in higher sensitivity and selectivity compared to the previously developed method [34]. No interfering compound was observed by analyzing the blank matrix (rearing water with the unicellular green alga and the yeast and without *Daphnia*), thus providing evidence that MF is secreted by *Daphnia*. Detection and quantitation limits of 0.03 and 0.08 ng/L were obtained, about five times lower than those previously reported [34], due to the reduced background noise (S1 Table). The developed method was characterized by a very good intermediate precision (RSD < 10%) and recovery rates in the 94.7(±1.4)-103.4(±2.8) % range (n = 10). A negligible matrix effect was observed: no significant difference (*p* > 0.05) was obtained by comparing the slope of the calibration curve obtained using standard solutions with that achieved by using the standard addition method. The enhanced detection and quantification capabilities were crucial for accurately assessing variations in endogenous MF concentrations released into the water across different experimental conditions. The developed SPME-GC-MS/MS method was therefore applied to monitor the release of MF by *D. pulex* under different stress conditions.

### *D. pulex* culturing

Life-table experiments were performed by using two clones, I and S, which differ in the sensitivity to the environmental stimuli leading to the production of male/ephippia and neckteeth phenotypes (S1 Appendix).

### Experiment 1: Effects of crowding due to a reduction of water volume and MK-801 addition on life-history traits and MF release

The effect of crowding due to a reduction of water volume and MK-801 addition (1µM) on the life cycle of *D. pulex* clone I and clone S was tested over two generations (G1 and G2) (S1 Fig).

In G1, no significant differences in the age at first reproduction were observed among treatments and between clones. Both clones showed a significant reduction in carapace length and in the number of neonates per female in Crow and MK treatments compared to control (Fig 1, panel A and S2 Table). Only the clone S produced males (7.8% of total juveniles) and ephippia (8.5% of total clutches) under crowding conditions.

**Fig 1:**
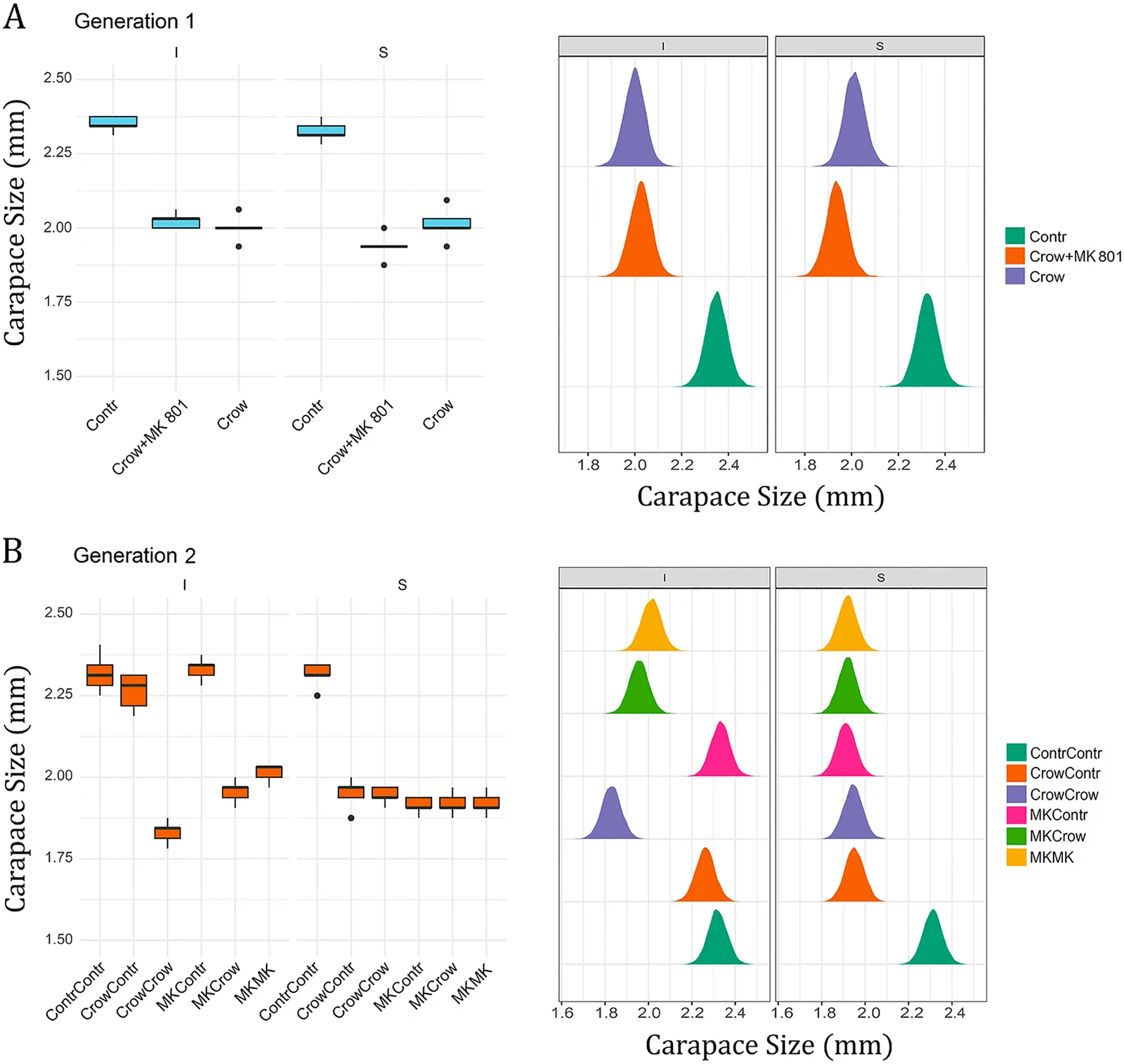
Carapace length for generation 1 and 2. Each panel depicts carapace length (mm) for G1 (Panel A) and G2 (Panel B). The left side illustrates the trait distribution, while the right side shows the posterior distribution estimated using Bayesian model for life table traits. Results were presented for clones I and S under different treatments.

As for G2, the credibility interval of the carapace length of 8-day-old females was significantly smaller under CrowCrow, MKCrow and MKMK treatments than in the ContrContr for both clones (Fig 1, S2 Table). However, a different recovery was assessed between the two clones: in clone I carapace length was recovered in G2 CrowContr and MKContr, whereas in clone S the size remains smaller than in control, even after recovery in G2 CrowContr and MKContr treatments (Fig 1, S2 Table). The age at first deposition did not differ among treatment and between clones (S2 Fig). A significant decrease in fecundity was observed for both clones when both generations were subjected to crowding with (CrowMK) or without (CrowCrow) exposure to MK-801 (Fig 2, panel A). The same behaviour was observed when the first generation was exposed to MK-801 and the second one was reared under crowding conditions without MK-801 (MKCrow) (Fig 2, panel B). However, when only G1 was subjected to crowding, fecundity significantly recovered in G2 under control conditions, regardless the exposure to MK-801 (CrowContr and MKControl). Clone S showed a higher recovery than clone I although fecundity was significantly lower than the control treatment (ContrContr).

**Fig 2:**
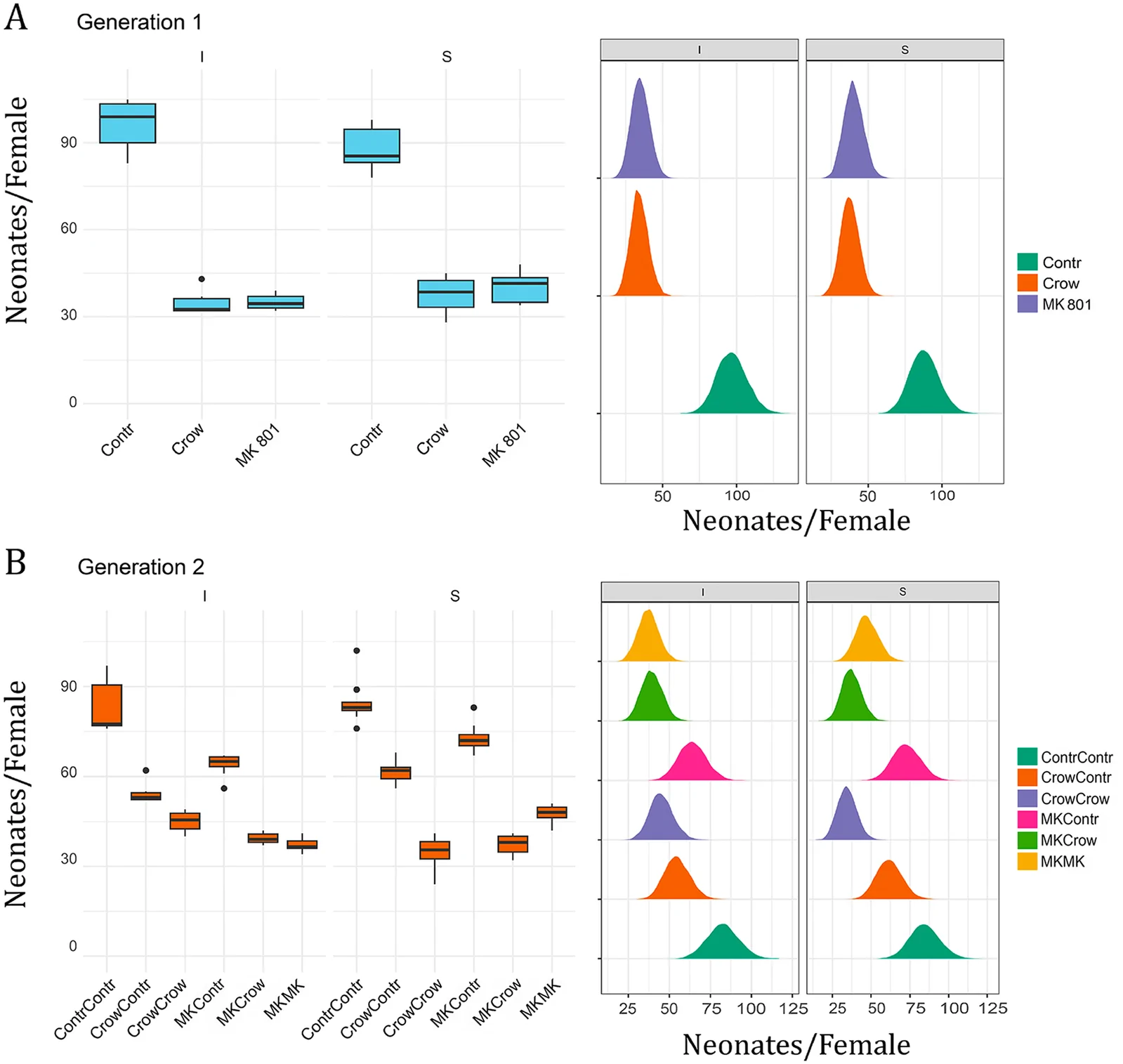
Neonates per female for both generations. Each panel depicts neonates per female for G1 (Panel A) and G2 (Panel B). The left side illustrates the trait distribution, while the right side shows the posterior distribution estimated using Bayesian model. Results were presented for clones I and S under different treatments (see Materials and Methods).

Only clone S produced male offspring and ephippia in CrowCrow and in MKCrow treatments, which also corresponded with the lowest levels of fecundity. The highest production of males and ephippia was recorded for CrowCrow with 25.7% of males and 14.0% of ephippia while the percentages decreased to values recorded in G1 subjected to crowding (Crow), with 8.3% of males and 6.4% of ephippia, when G2 was subjected to crowding but not exposed to MK-801 (i.e., MKCrow) (Fig 3).

**Fig 3:**
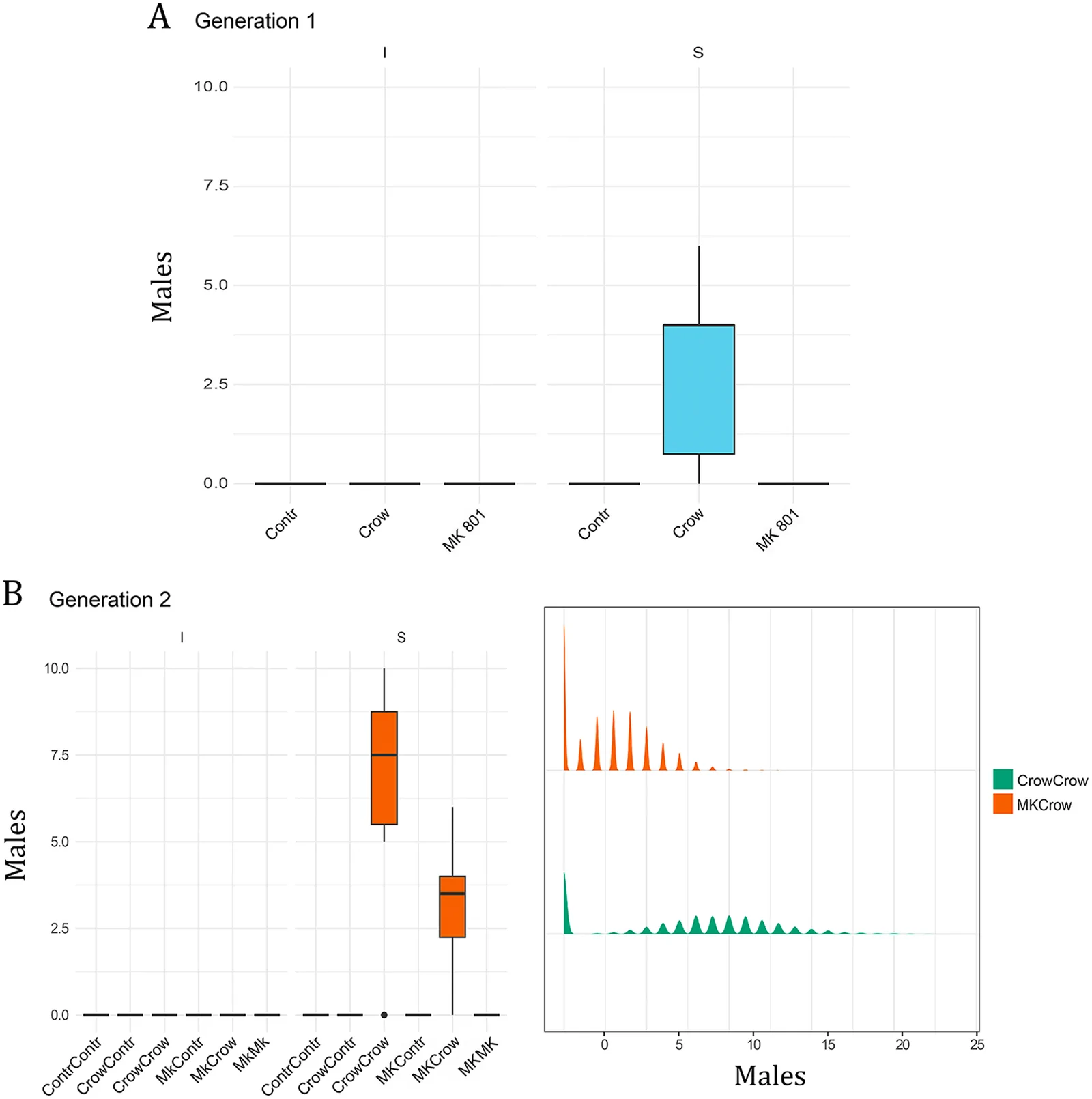
Number of males for Generation 1 and Generation 2. Panel A illustrates the trait distribution for G1, while panel B shows the posterior distribution for G2 estimated using Bayesian model. Results are presented for clones I and S under different treatments (see Materials and Methods).

The highest MF concentration was detected in controls, whereas a significant, progressive reduction was observed under crowding condition and in presence of MK-801 (S3 Fig). In G2 the MF concentration released was lower than in G1; a decrease was observed when daphnids were subjected to crowding for two generation (CrowCrow) and values lower than LOQ were observed when daphnids were subjected to MK-801 in the first generation (MKContr, MKCrow and MKMK). As for clone S, MF concentration was higher than clone I CrowCrow. For both clones, an increase of MF levels was observed after recovery from crowding (CrowControl).

### Experiment 2: Effects of crowding due to increasing density and seasonal condition on life-history traits and MF release

The effect of crowding due to increasing density (and consequently a lowered availability of food per individual) and seasonal variation (early and late spring) were also evaluated on the MF release.

Mortality was negligible. The mean carapace length of 8-day-old females for both clones was lower under the conditions simulating early spring than those simulating the late spring (Fig 4 panel A). In clone I dimensions were not significantly affected by population density. In clone S, the difference in body size between treatments under the two seasonal conditions progressively decreased with increasing density and was no longer evident at densities 4 and 5, as mean body size decreased under late-spring conditions and increased under early-spring conditions. Under early spring conditions, females of both clones reach sexual maturity (age at first deposition) in a longer time (8–10 days) than under late spring conditions (6–7 days) (N = 86, median test, p < 0.001). No significant difference was observed among densities. In the treatment simulating early spring short photoperiod, due to the delayed development, the experiment was extended from 21 to 23 days.

**Fig 4:**
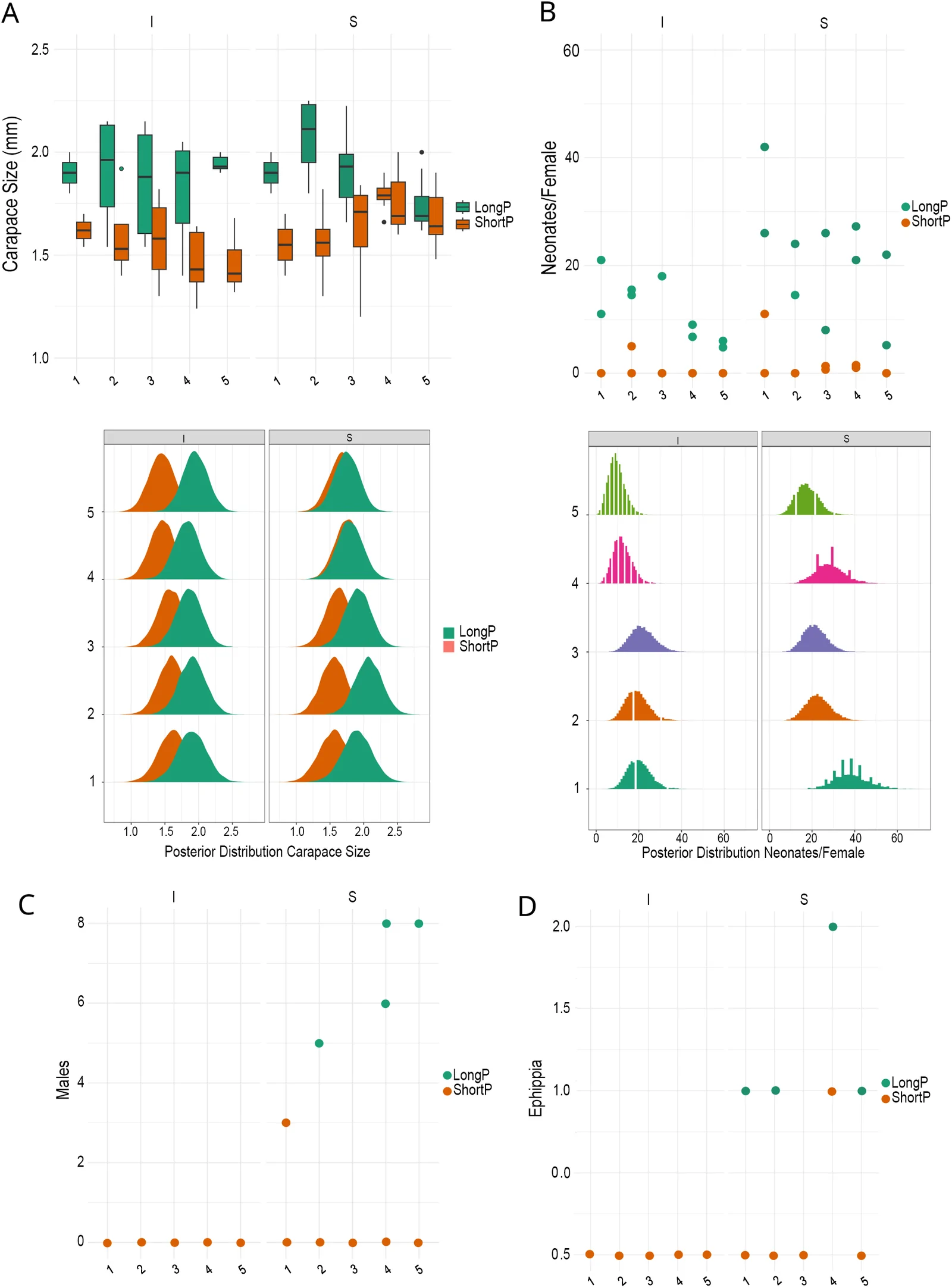
Life table variables for both clones under different photoperiods. Experiment 2, each panel represents life table variables for clone I and clone S at different densities in ShortP and LongP environmental conditions: carapace length (A), Neonates per female (B), Males (C) and ephippia (D). In panel B (bottom), neonates per female posterior distribution was estimated on LongP.

A total of 927 neonates were produced, and, in both clones, fecundity was particularly lower in conditions simulating early spring (short photoperiod, ShortP) than late spring (long photoperiod, LongP) (Fig 4). For clone I, fecundity increased up to density 3 and then decreased, whereas for clone S, production increased up to density 4 before decreasing. The average number of neonates per female showed great variability, especially for clone S (Fig 4, panel B).

Focusing on the conditions simulating late spring long photoperiod, fertility was significantly higher in the S clone than in clone I and it was not affected by density (Fig 4, panel B). Under both experimental conditions, irrespective to the density, only clone S females produced males and ephippia (Fig 4, panels C and D).

Five different morphotypes were observed: M0, M1, M2, M3 and M4. Altogether, the frequency of neckteethed first-instar juveniles was higher in clone I than in clone S and morphotype M4 was only identified among the offspring of clone I (S4 Fig). Considering only the late spring condition, the one with the highest neonate production, significant differences in the percentage of neckteethed juveniles were observed between clones and among densities (χ^2^_76_ = 228.85, *p*-value < 0.0001). For both clones, an increase in the frequency of morphotypes with two or more neckteeth was positively related with the increasing density (S4 Fig).

As for MF, at density 1, concentrations below 1 ng/L were observed only in clone S under late spring conditions (S5 Fig). At densities 2 and 5, MF levels up to 9.7 ng/L were detected in the water of both clones under early or late spring conditions, except for clone I at 8 and 21 days in the LongP and at day 21 in the ShortP (S5 Fig). Considering the values per female, the release was higher in early than in late spring and clone S released more MF than clone I (S6 Fig).

To relate MF concentration and the production of male and ephippia, only data from clone S were considered. The concentration of MF released in water per female decreased with the increasing of percentage of males (parameters: a = 6.46, b = 0.19; AIC = 31.22) and ephippia (parameters: a = 4.36, b = 0.02; AIC = 35.69) (Fig 5, panels A and B).

**Fig 5:**
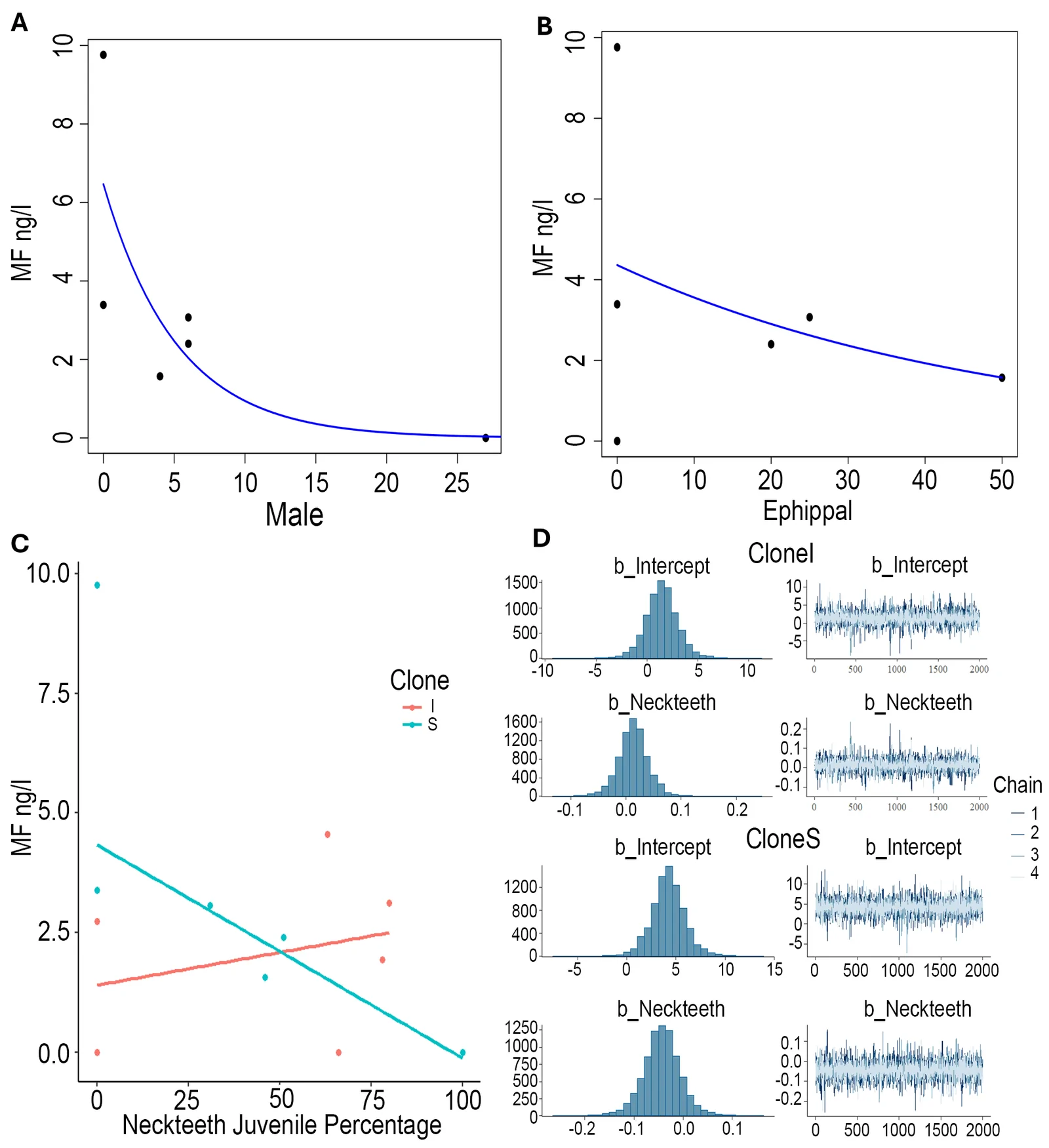
Associations between MF concentration and reproductive/defensive traits in clones I and S. Relationship between percentage of males (panel A), ephippia (panel B) and total MF concentration per female in clone S. The blue line represents non-linear least squares fit of the decay function. Linear relationship between percentage of neckteeth morphotypes and total MF concentration per female in the I and S clones (panel C). For both clones, the Bayesian posterior distributions of the intercept (top) and slope (bottom) of the linear model are reported (panel D).

The relationship between the concentration of MF released in water and the percentage of neckteeth morphotypes is different. In clone S, the values were inversely related (mean slope value of −0.04), whereas in clone I a slight positive correlation was observed (mean slope value of 0.01). (Fig 5, panels C and D).

## Discussion

In the present study, we hypothesized that changes in environmental conditions, such as crowding, temperature, and photoperiod, may modulate the release of endogenous MF into the water, thereby influencing the production of males, ephippia, and neckteeth. Two clones, I and S, characterized by different propensities to produce males, ephippia, and neckteeth phenotypes, were considered. The presence of MF in water in which *D. pulex* had been reared was previously reported [34].

Body-size responses provided additional evidence of clone-specific sensitivity to environmental conditions. Crowding induced by reducing the available water volume decreased body size and fecundity in both clones. Following recovery from crowding in G2, body-size recovery was more pronounced in clone I than in clone S, whereas the opposite pattern was observed for fecundity. In the population-density experiment, seasonal conditions had a clear effect on body size, with smaller females under early-spring conditions. In clone S, the difference between early- and late-spring conditions decreased at the highest densities, suggesting that increasing density reduced the effects associated with seasonal conditions. No significant density effect was detected in clone I, although opposite descriptive trends were observed under short- and long-photoperiod conditions. Overall, these results indicate different patterns of phenotypic plasticity between the two clones.

In the population-density experiment, increasing the number of individuals while maintaining a constant food concentration, reduced food availability per individual. Population density and per-capita food availability therefore covaried. Therefore, the response to a combined condition involving increased density, greater interaction among individuals, and reduced food availability per individual was considered. These conditions are based on previous studies showing that high density under high-food conditions produced on the same clones a greater reduction in fecundity than food limitation under low-density conditions and more strongly promoted male and ephippia production in clone S [35]. The release of MF by the *D. pulex* clones was therefore evaluated under this combined density–food availability condition and under two environmental regimes simulating the beginning and end of spring, when natural populations are in the exponential growth phase. No signal was detected in blank rearing medium samples. MF concentrations above the LOD were detected in the water of all treatments, although at density 1 it was released in detectable amounts only by females of clone S under late spring conditions.

In response to environmental stimuli, under early spring conditions, body size was smaller than that of females maintained under late spring conditions in both clones, whereas the opposite trend was observed for age at sexual maturity. Assuming that only adult females produce endogenous MF, the developmental delay could be related to the low or undetectable MF concentrations observed at density 1 on day 8 under early spring conditions.

Clone S produced males and ephippia under both early and late spring conditions. The highest percentage of males (26%) was observed under early spring treatment at density 1, corresponding to the only samples of clone S in which MF could not be detected in the breeding medium. In this context, MF concentration per female decreased as the percentages of males and ephippia increased. Clone S produced fewer neckteeth morphotypes than clone I, and the proportion of neckteethed morphotypes increased with the reduction of endogenous MF released into the water by each female. These results are consistent with the observation that clone S is more sensitive to density and intraspecific interactions than clone I. Clone S responded to crowding by reducing the release of MF, which could be redirected through the haemolymph to specific peripheral target cells, where it regulates the expression of male sex-determining genes and the production of males, ephippia, and neckteeth, as previously reported [35].

The ability of clone S to produce males even in the absence of exogenous MF may indicate that it is more sensitive to environmental stimuli than clone I and/or that a lower endogenous MF concentration is sufficient to activate the Met receptor [35]. The relationship between male and ephippia production and MF concentration may be linked to the different reproductive phases of cyclical parthenogenesis. MF is considered the main circulating hormonal regulator of a series of morphogenetic events in crustacean development, and it is necessary for larval maturation and metamorphosis in *Drosophila melanogaster* [3,38,40]. Jones et al. observed several alterations in larval development, metabolism, and morphogenesis after inhibiting the production of circulating farnesoid hormones [41]. One of the most evident effects was melanisation of the tracheal trunks. In *Daphnia,* physiological formation of the ephippium involves melanisation of the incubator chamber, which generally follows male production: melanisation should be linked to variations in MF concentration.

In clone I, increasing density and the consequent reduction in food availability per individual reduced fecundity but did not induce the production of males or ephippia. Confirming previous results [18], clone I showed a higher propensity to produce neckteethed juveniles than clone S and produced a high percentage of neckteethed juveniles even under crowding conditions. In clone I, the proportion of neckteethed morphotypes was not related to a significant reduction in endogenous MF released into the water by single females. Miyakawa et al. suggested that neckteeth production is under the control of the canonical JH signalling pathway, because JH pathway-related genes, *JHAMT* and *Met*, are upregulated in response to kairomones [12]. Clone I was less sensitive to crowding than clone S, exhibiting the highest production of neckteethed juveniles while maintaining the release of endogenous MF into the water. This could be explained by considering that a low endogenous MF concentration is sufficient to activate genes regulating neckteethed juvenile production [38].

Although *Daphnia* are phylogenetically more closely related to insects [42], in *D. pulex* MF is considered the native JH because of the lack of the enzyme CYP15A1, which is involved in JH III synthesis [10,14,15]. Toyota et al. confirmed that MF is the native JH in *D. pulex* and identified the enzyme JHAMT as responsible for its endogenous synthesis, although they were not able to quantify MF concentrations in the haemolymph of *D. pulex* [9]. According to our results, the concentration of MF released into the water by *D. pulex*, in the absence of other possible sources, ranged from 1.56 to 12.05 ng/L.

These findings provide indirect evidence of MF release into the rearing water by *D. pulex*. The observed associations between MF concentrations in the rearing medium and life-history traits, however, do not demonstrate a direct mechanistic relationship. In particular, the lower MF concentrations observed under crowding cannot be interpreted as evidence of reduced endogenous MF production, because the concentration measured in the medium may reflect the combined effects of release, accumulation, degradation, adsorption and uptake. Further studies will be aimed at measuring MF within the organisms, its release in the surrounding medium and the influencing factors to determine the mechanisms underlying these variations.

Finally, this study does not aim to demonstrate a causal endocrine role of MF in sex determination or phenotypic plasticity in *Daphnia*. Rather, it provides quantitative, environmentally relevant evidence of MF release into the aquatic medium, contributing indirect and ecological data to the ongoing debate on the role of JH‑like signalling pathways in cladocerans. Given the central ecological role of *Daphnia* as a keystone species in freshwater food webs, improving our understanding of the biochemical mediators underlying its stress responses is essential for both ecotoxicological assessment and ecosystem‑level studies.

## Supporting information

S1 Appendix Materials and methods integration. (PDF)

S1 Table Precision and trueness of the SPME-GC-MS/MS method. (PDF)

S2 Table Credibility interval for each trait, for both clones in Generation 1 and Generation 2. (PDF)

S1 Fig Schematic representation of the first experimental design. Contr: control condition; Crow: crowding condition; MK: MK-801 treatment.
(PDF)

S2 Fig Age at first deposition in Generation 1 (A) and Generation 2 (B). Contr: control; ContrContr: G2 control daphnids born to G1 control females; CrowContr: G2 control daphnids born to G1 females exposed to crowding; CrowCrow: G1 and G2 exposed to crowding; MKContr: G2 control daphnids born to G1 females exposed to MK-801; MKCrow: G2 daphnids exposed to crowding born to G1 females exposed to MK-801: MKMK: both G1 and G2 exposed to 1.0 µM MK-801.
(PDF)

S3 Fig Concentration of MF released in the growth medium by clones I and S of *D. pulex.* *G1* - C: control; CR: crowding; CR + MK801: crowding + MK801. *G2*: CC: control daphnids born to G1 control females; CRC control daphnids born to G1 females exposed to crowding; CRCR: both generations exposed to crowding; MKC: control daphnids born to G1 females exposed to MK-801; MKCR: daphnids exposed to crowding born to G1 females exposed to MK-801; MKMK: both generations exposed to MK −801.
(PDF)

S4 Fig Distribution of morphotypes in clones I and S across population density and photoperiod conditions. Percentage of five different morphotypes (M0-M4) in clone I and clone S at different population densities under two environmental conditions: early spring short photoperiod (ShortP) and late spring long photoperiod (LongP).
(PDF)

S5 Fig Concentration of MF released at different population densities. Concentration of MF released at densities 1, 2 and 5 at day 8, day 14 and day 21 by clones I and S under different experimental conditions (IL: clone I at late spring long photoperiod, IS: clone I at early spring short photoperiod, SL: clone S at late spring long photoperiod SS: clone S at early spring short photoperiod).
(PDF)

S6 Fig Total MF concentration per female at different population densities. Total MF concentration normalized considering the number of females at different densities for clones I and S in the early (S) and late (L) spring photoperiod.
(PDF)

S7 Fig SPME-GC-MS/MS chromatogram and spectrum of MF in a real sample. SPME-GC-MS/MS chromatogram (top) and MS/MS spectrum (bottom) of MF obtained from clone S, at density 2, under late spring conditions after 14 days.
(PDF)

S8 Fig Graphical abstract. (TIFF)
