# Supplementary material for: Environmental modulation of methyl farnesoate release in two clones of Daphnia pulex differing in the sensitivity to exogenous stimuli
Source: PLoS One. 2026 Oct 27;21(10):e0361189. doi: 10.1371/journal.pone.0361189 (PMC13712590; doi:10.1371/journal.pone.0361189)
Supplement: Supplementary file 1 [file pone.0361189.s001.pdf]

S1 Appendix. Materials and Methods Integration.

Clones

The two clones (I and S) were isolated from zooplankton samples collected from the pond Bodrio del pastore III located in northern Italy (45° 00’ 05” N 10° 19’ 26” E) and a village pond Sedlec (Czech Republic), 50° 26' 21" N 14° 36' 29 E) [28] previously selected according to Suppa et al. [18]. The two clonal lineages were determined as European D. pulex based on morphological and molecular analyses [29] and are genetically different [18].

Method Validation

Detection (y_D) and quantitation (y_Q) limits were expressed as signals based on the mean blank (x_b) and the standard deviation of blank responses (s_b) as follows: $y_D = x_b + 3s_b$ and $y_Q = x_b + 10\ s_b$. The value of x_b and s_b were calculated performing ten blank measurements. Detection (LOD) and quantitation limits (LOQ) were obtained by projection of the corresponding signals y_D and y_Q through a calibration plot y=f(x) onto the concentration axis.

Calibration was performed using an external calibration in a LOQ-40 ng/L range (six concentration levels, three replicated measurements for each level) using methyl decanoate as IS at the concentration of 20 ng/L.

Repeatability and intermediate precision were calculated in terms of relative standard deviations (RSD%) at three concentration levels (LOQ, 5 and 20 ng/L, six replicated measurements per level). Intermediate precision was estimated over three days verifying homoscedasticity of data and performing the analysis of variance (ANOVA) at the confidence level of 95%. Trueness was calculated in terms of recovery rate (RR%) as follows:

$RR\% = c_{exp}/c_{spike} \times 100$, where c_{exp} is the measured concentration, and c_{spike} is the concentration obtained by spiking the blank sample. Recovery rate values were assessed by performing ten replicated measurements per level at the same levels used for the evaluation of precision.

Experiment 1: combined and transgenerational effects of crowding and MK-801 on life-history traits of *D. pulex* clones

G1 - Ten female juveniles per clone per treatment (control, crowding and Crow + MK-801; 3 treatments × 2 clones × 10 replicates = 60 females) were individually reared and fed with a mixture of *P. subcapitata* and *S. cerevisiae*, each at the final concentration of 1.5×10^5 cells/ml. In each treatment, medium and food were renewed three times a week.

Finally, daphnids were separated by mesh filtration and the water samples (one vial per clone per treatment) where the adult females were maintained for the last 48 hours, were stored at -20°C before the SPME-GC-MS/MS analysis [33].

G2 - Fifteen female juveniles per clone per treatment (2 treatments × 2 clones × 2 replicates = 120 females) were reared in 50 ml at densities of 1, 2, 3, 4, or 5 females per beaker in two experimental conditions. The first was intended to recreate an early spring environmental condition and therefore the organisms were kept in a thermostatically controlled chamber at 16°C with a photoperiod of 12:12 L:D hours (early spring short photoperiod, S). The second one was intended to recreate a late spring condition, and the organisms were kept in a thermostatically controlled chamber maintained at 20°C with a photoperiod of 14:10 L:D (late spring long photoperiod, L). The females were reared in NW and fed with 1.5×10^5 cells/ml of *P. subcapitata* and *S. cerevisiae* yeast.

Two replicates for each density were performed using a total of 30 females for each clone and each experimental condition (16°C 12:12 L:D h, 20°C 14:10 L:D h) (30 females × 2 clones × 2 conditions = 120 females). The experiment lasted 21 days. In each treatment, NW medium and food were renewed three times a week.

Body size (as carapace length from the top of the head to the base of the tail spine) was measured on the 8th day of treatment); age at maturity (the age at which the first eggs are deposited into the brood chamber) and fecundity (total number of alive offspring per female) were recorded three times a week. Juveniles were removed and classified according to their gender and morphotype (M0 - no teeth, M1 - one tooth, M2 - two teeth, M3 - three teeth and M4 – four teeth) [18] using a CLS 50× microscope (Leica CLS 50× microscope; Leica, Mannheim, Germany).

Experiment 2: effects of density and seasonal condition on life-history traits and MF release by *D. pulex* clones

Fifteen female juveniles per clone per treatment (2 treatments × 2 clones × 2 replicates = 120 females) were reared in 50 ml at densities of 1, 2, 3, 4, or 5 females per beaker in two experimental conditions. The first was intended to recreate an early spring environmental situation and therefore the organisms were kept in a thermostatically controlled chamber at 16°C with a photoperiod of 12:12 L:D hours (early spring short photoperiod, S). The second one was intended to recreate a late spring condition, and the organisms were kept in a thermostatically controlled chamber maintained at 20°C with a photoperiod of 14:10 L:D (late spring long photoperiod, L). The females were reared in NW and fed with 1.5 × 105 cells/ml of *P. subcapitata* and *S. cerevisiae* yeast.

Two replicates for each density were performed using a total of 30 females for each clone and each experimental condition (16°C 12:12 L:D h, 20°C 14:10 L:D h) (30 females × 2 clones × 2 conditions = 120 females). The experiment lasted 21 days. In each treatment, NW medium and food were renewed three times a week.

Body size (as carapace length from the top of the head to the base of the tail spine) was measured on the 8th day of treatment); age at maturity (the age at which the first eggs are deposited into the brood chamber) and fecundity (total number of alive offspring per female) were recorded three times a week. Juveniles were removed and classified according to their gender and morphotype (M0 - no teeth, M1 - one tooth, M2 - two teeth, M3 - three teeth and M4 – four teeth) [18] using a CLS 50× microscope (Leica CLS 50× microscope; Leica, Mannheim, Germany).
