# Supplementary material for: Environmental modulation of methyl farnesoate release in two clones of Daphnia pulex differing in the sensitivity to exogenous stimuli
Source: PLoS One. 2026 Oct 27;21(10):e0361189. doi: 10.1371/journal.pone.0361189 (PMC13712590; doi:10.1371/journal.pone.0361189)
Supplement: Supplementary file 2 [file pone.0361189.s002.pdf]

S1Table. Precision and trueness of the SPME-GC-MS/MS method.

Slope of Calibration Curve	$5.13 \pm 0.23 \times 10^{-3}$		
LOD (ng/L)	0.03		
LOQ (ng/L)	0.08		
	Concentration Level (ng/L)		
	LOQ	5	20
Repeatability RSD%	9.2	7.3	5.6
Intermediate Precision RSD%	9.7	6.8	5.8
Trueness RSD% (±st.dev.)	94.7±1.4	101.6±1.9	103.4±2.8

calibration curve y = bx.
