# Supplementary material for: Environmental modulation of methyl farnesoate release in two clones of Daphnia pulex differing in the sensitivity to exogenous stimuli
Source: PLoS One. 2026 Oct 27;21(10):e0361189. doi: 10.1371/journal.pone.0361189 (PMC13712590; doi:10.1371/journal.pone.0361189)
Supplement: Supplementary file 3 [file pone.0361189.s003.pdf]

S2 Table. Credibility interval for each trait, for both clones in Generation 1 and Generation 2.

	Carapace Length (mm)		
	**1 Generation**		
	**2.5%**	**97.5%**	***Clones***
Control	2.25	2.44	I
Crowding	1.90	2.09	
CrowMK801	1.93	2.12	
Control	2.23	2.42	S
Crowding	1.92	2.10	
CrowMK801	1.84	2.03	
	**2 Generation**		
	**2.5%**	**97.5%**	
ContrContr	2.23	2.41	I
CrowContr	2.17	2.35	
CrowCrow	1.74	1.92	
MKContr	2.24	2.42	
MKCrow	1.87	2.04	
MKMK	1.92	2.10	
ContrContr	2.22	2.40	S
CrowContr	1.86	2.04	
CrowCrow	1.85	2.03	
MKContr	1.82	2.00	
MKCrow	1.83	2.00	
MKMK	1.83	2.00	
	Neonates per female		
	**1 Generation**		
	**2.5%**	**97.5%**	
Control	78	118	I
Crowding	23	47	
CrowMK801	24	47	
Control	69	108	S
Crowding	26	51	
CrowMK801	28	54	
	**2 Generation**		
	2.5%	97.5%	
ContrContr	65	102	I
CrowContr	40	70	
CrowCrow	32	60	
MKContr	48	81	
MKCrow	27	53	
MKMK	25	50	
ContrContr	67	104	S
CrowContr	46	79	
CrowCrow	23	47	
MKContr	56	91	
MKCrow	25	51	
MKMK	34	62	

Males			
**2 Generation**			
	2.5%	97.5%	
CrowCrow	0	17	
MKCrow	0	10	S
