# Supplementary material for: Environmental modulation of methyl farnesoate release in two clones of Daphnia pulex differing in the sensitivity to exogenous stimuli
Source: PLoS One. 2026 Oct 27;21(10):e0361189. doi: 10.1371/journal.pone.0361189 (PMC13712590; doi:10.1371/journal.pone.0361189)
Supplement: Supplementary file 4 [file pone.0361189.s004.pdf]

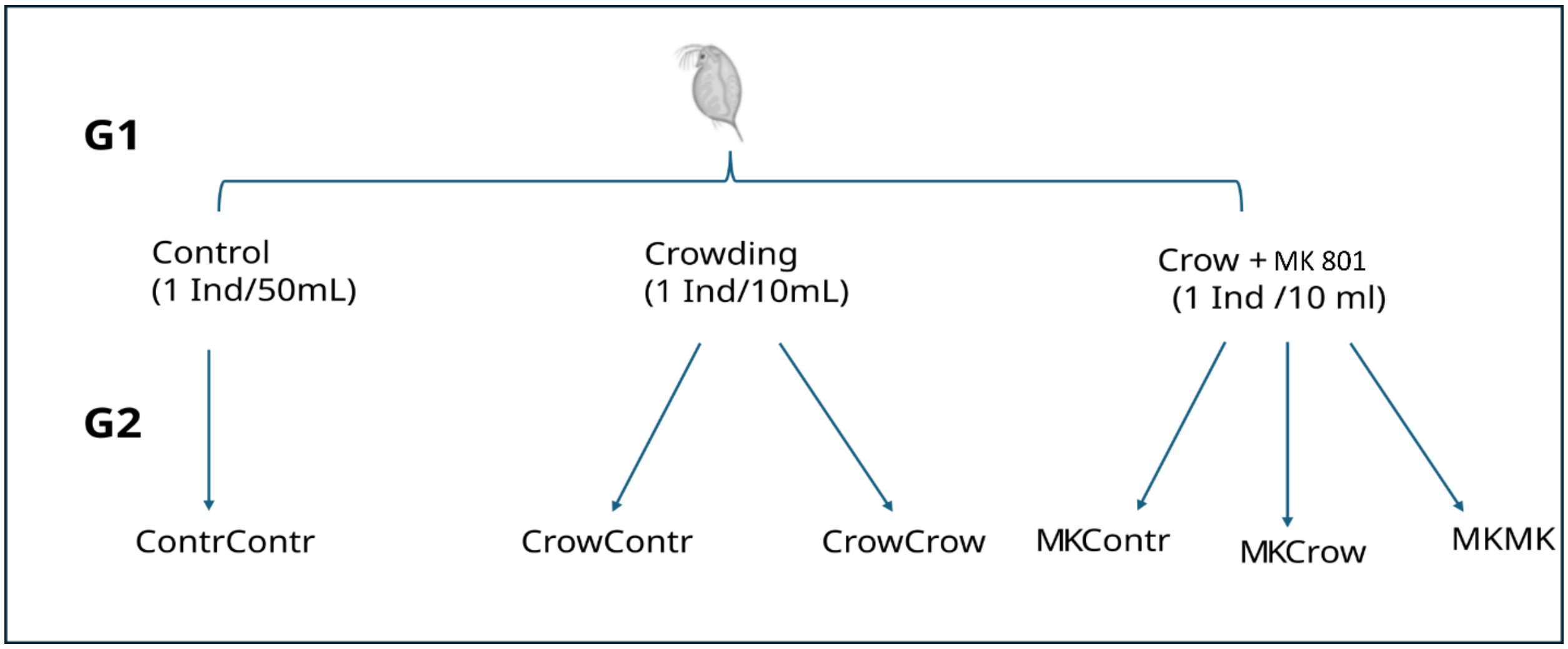

S1 Fig. Schematic representation of the first experimental design. Contr: control condition; Crow: crowding condition; MK: MK-801 treatment.
