# Supplementary material for: Environmental modulation of methyl farnesoate release in two clones of Daphnia pulex differing in the sensitivity to exogenous stimuli
Source: PLoS One. 2026 Oct 27;21(10):e0361189. doi: 10.1371/journal.pone.0361189 (PMC13712590; doi:10.1371/journal.pone.0361189)
Supplement: Supplementary file 5 [file pone.0361189.s005.pdf]

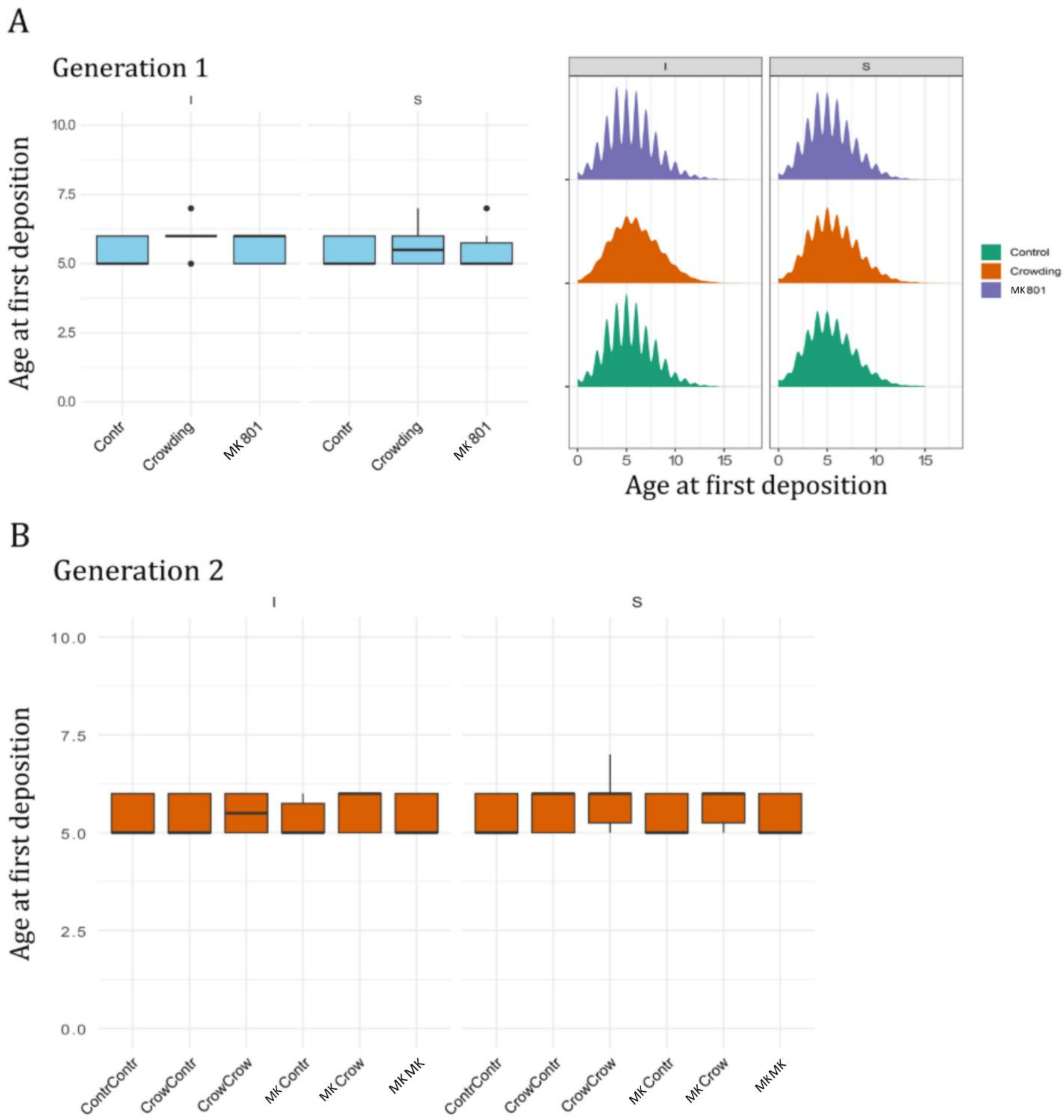

S2 Fig. Age at first deposition in Generation 1 (A) and Generation 2 (B). Contr: control; ContrContr: G2 control daphnids born to G1 control females; CrowContr: G2 control daphnids born to G1 females exposed to crowding; CrowCrow: G1 and G2 exposed to crowding; MKContr: G2 control daphnids born to G1 females exposed to MK-801; MKCrow: G2 daphnids exposed to

crowding born to G1 females exposed to MK-801: MKMK: both G1 and G2 exposed to 1.0 μM MK-801.
