# Supplementary material for: Environmental modulation of methyl farnesoate release in two clones of Daphnia pulex differing in the sensitivity to exogenous stimuli
Source: PLoS One. 2026 Oct 27;21(10):e0361189. doi: 10.1371/journal.pone.0361189 (PMC13712590; doi:10.1371/journal.pone.0361189)
Supplement: Supplementary file 6 [file pone.0361189.s006.pdf]

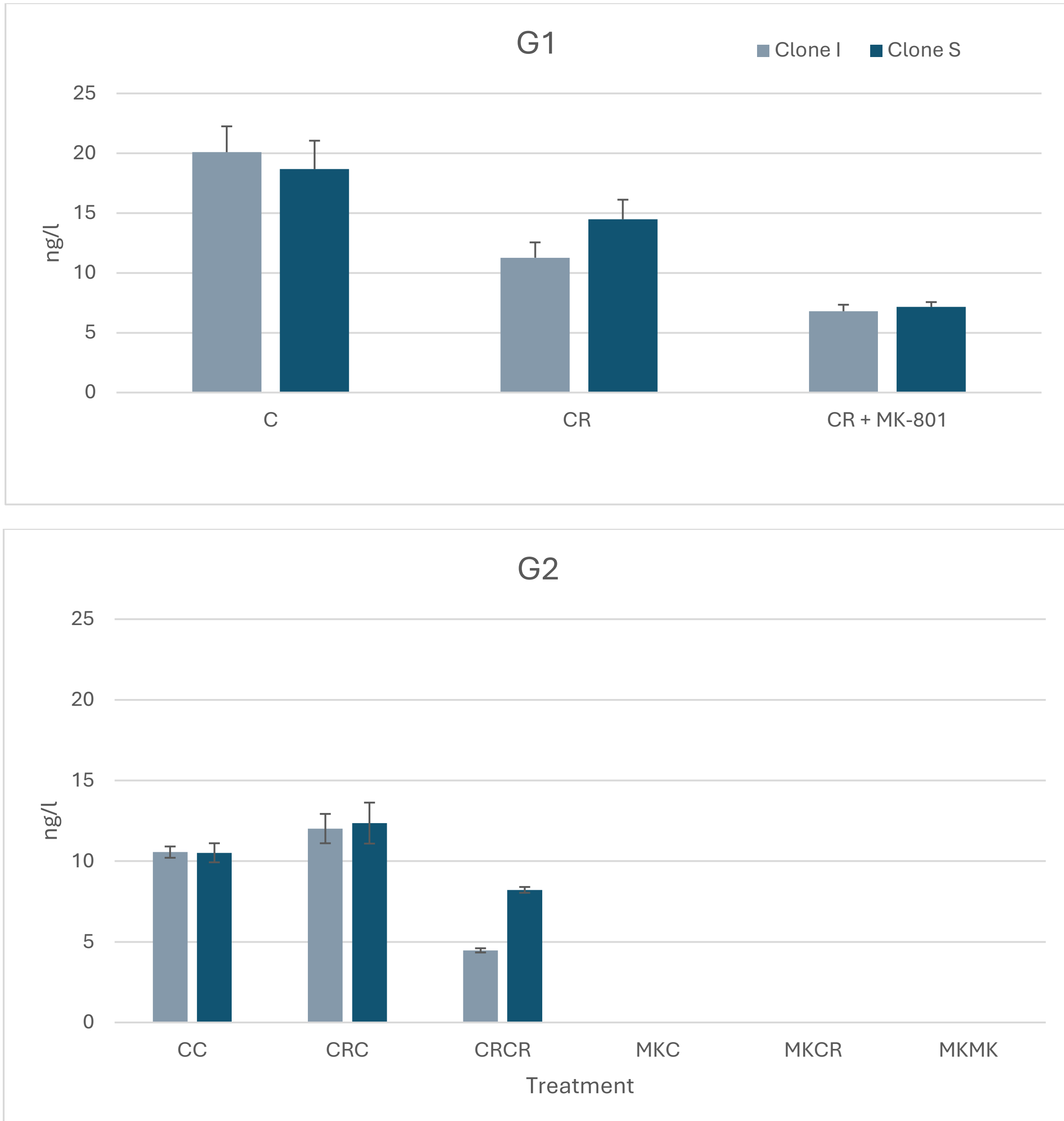

S3 Fig. Concentration of MF released in the growth medium by clones I and S of *D. pulex*.

G1 - C: control; CR: crowding; CR+MK801: crowding + MK801. *G2*: CC: control daphnids born to G1 control females; CRC control daphnids born to G1 females exposed to crowding; CRCR: both generations exposed to crowding; MKC: control daphnids born to G1 females exposed to MK-801; MKCR: daphnids exposed to crowding born to G1 females exposed to MK-801; MKMK: both generations exposed to MK -801.
