# Supplementary material for: Environmental modulation of methyl farnesoate release in two clones of Daphnia pulex differing in the sensitivity to exogenous stimuli
Source: PLoS One. 2026 Oct 27;21(10):e0361189. doi: 10.1371/journal.pone.0361189 (PMC13712590; doi:10.1371/journal.pone.0361189)
Supplement: Supplementary file 7 [file pone.0361189.s007.pdf]

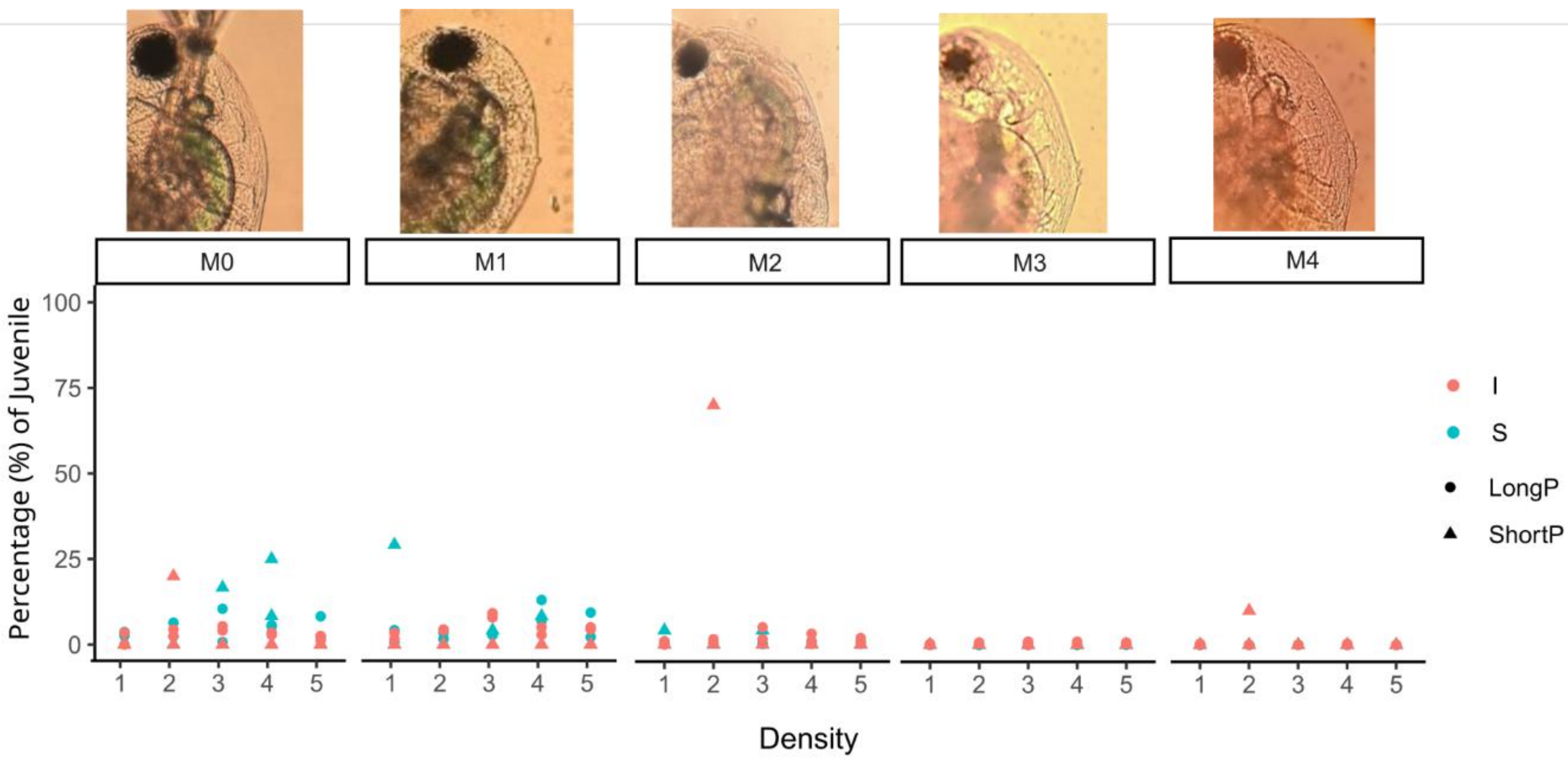

S4 Fig. Distribution of morphotypes in clones I and S across population density and photoperiod conditions. Percentage of five different morphotypes (M0-M4) in clone I and clone S at different population densities under two environmental conditions: early spring short photoperiod (ShortP) and late spring long photoperiod (LongP).
