# Supplementary material for: Environmental modulation of methyl farnesoate release in two clones of Daphnia pulex differing in the sensitivity to exogenous stimuli
Source: PLoS One. 2026 Oct 27;21(10):e0361189. doi: 10.1371/journal.pone.0361189 (PMC13712590; doi:10.1371/journal.pone.0361189)
Supplement: Supplementary file 8 [file pone.0361189.s008.pdf]

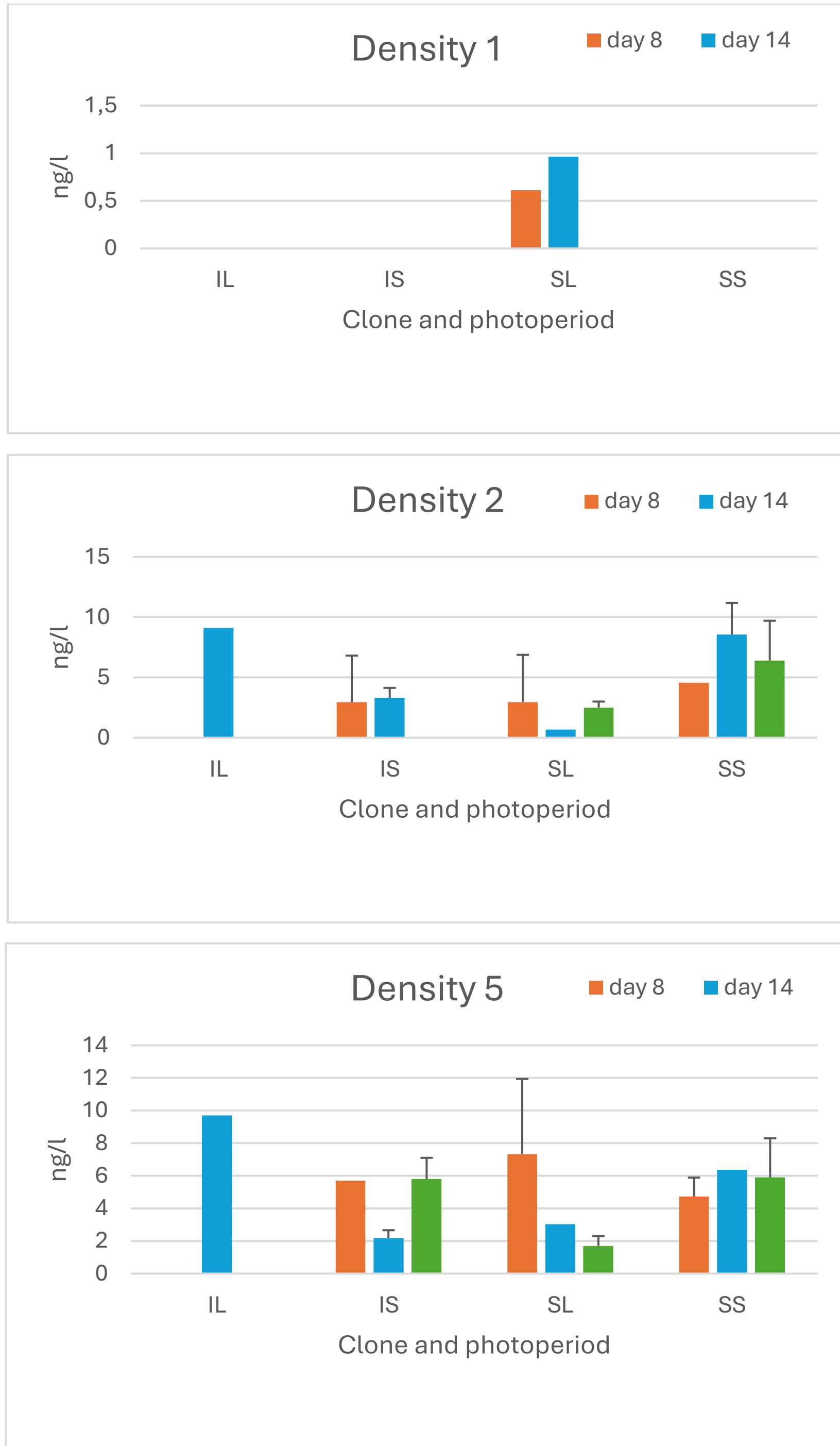

S5 Fig. Concentration of MF released at different population densities. Concentration of MF released at densities 1, 2 and 5 at day 8, day 14 and day 21 by clones I and S under different experimental conditions (IL: clone I at late spring long photoperiod, IS: clone I at early spring

short photoperiod, SL: clone S at late spring long photoperiod SS: clone S at early spring short photoperiod).
