# Supplementary material for: Environmental modulation of methyl farnesoate release in two clones of Daphnia pulex differing in the sensitivity to exogenous stimuli
Source: PLoS One. 2026 Oct 27;21(10):e0361189. doi: 10.1371/journal.pone.0361189 (PMC13712590; doi:10.1371/journal.pone.0361189)
Supplement: Supplementary file 9 [file pone.0361189.s009.pdf]

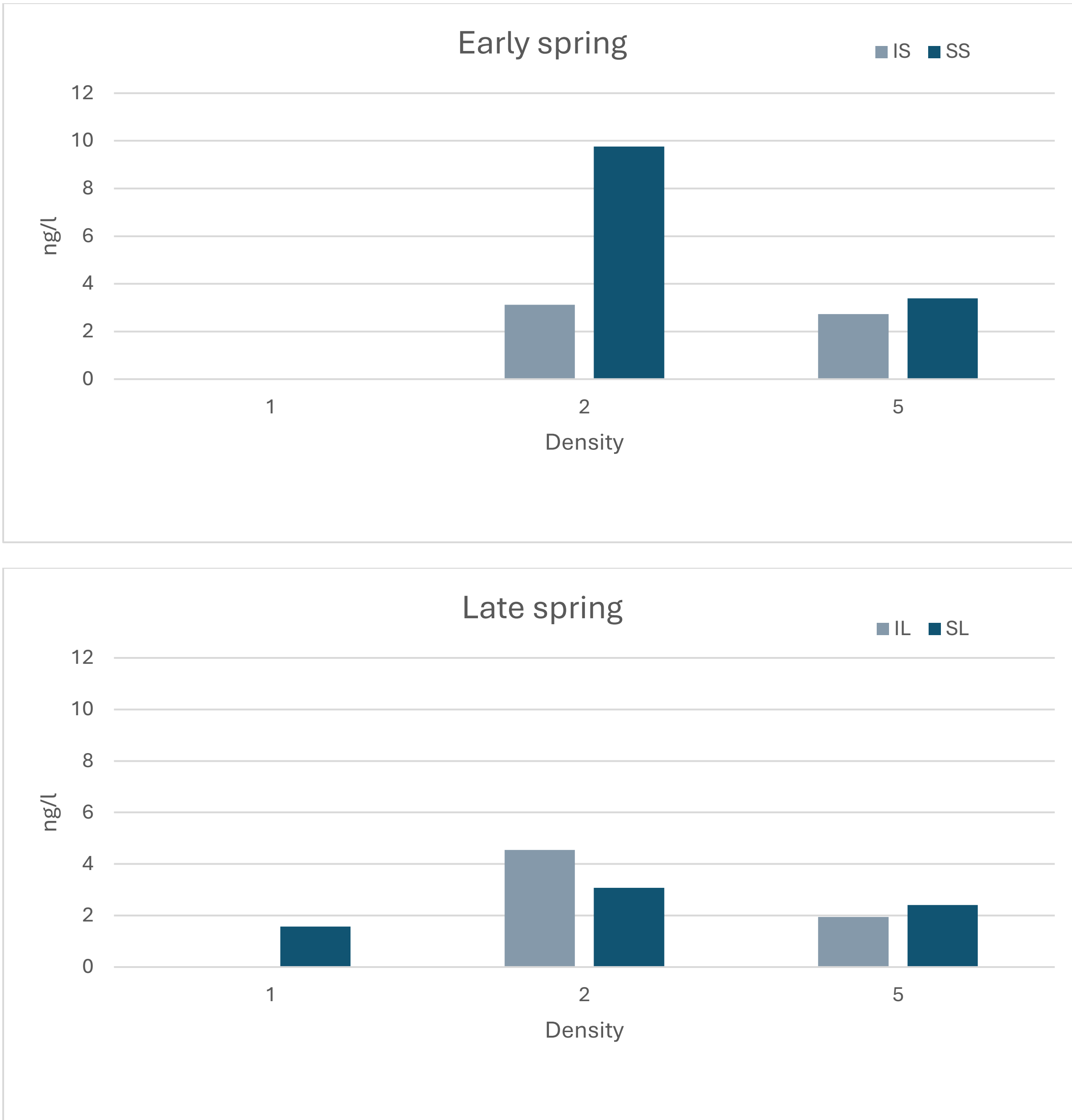


**S6 Fig. Total MF concentration per female at different population densities**. Total MF concentration normalized considering the number of females at different densities for clones I and S in the early (S) and late (L) spring photoperiod.
