# Supplementary material for: Environmental modulation of methyl farnesoate release in two clones of Daphnia pulex differing in the sensitivity to exogenous stimuli
Source: PLoS One. 2026 Oct 27;21(10):e0361189. doi: 10.1371/journal.pone.0361189 (PMC13712590; doi:10.1371/journal.pone.0361189)
Supplement: Supplementary file 10 [file pone.0361189.s010.pdf]

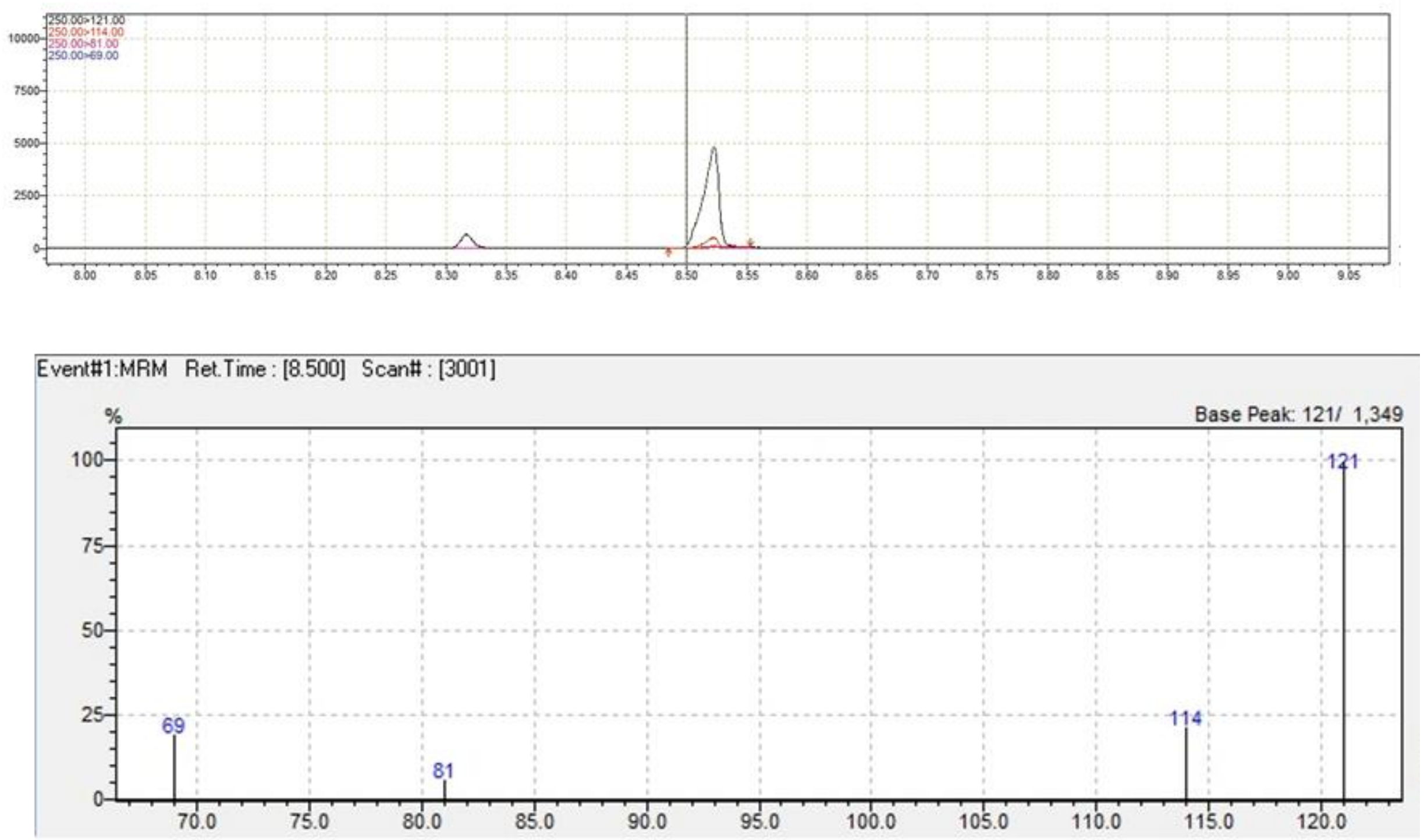

S7 Fig. SPME-GC-MS/MS chromatogram and spectrum of MF in a real sample. SPME-GC-MS/MS chromatogram (top) and MS/MS spectrum (bottom) of MF obtained from clone S, at density 2, under late spring conditions after 14 days.
