# Supplementary material for: Environmental modulation of methyl farnesoate release in two clones of Daphnia pulex differing in the sensitivity to exogenous stimuli
Source: PLoS One. 2026 Oct 27;21(10):e0361189. doi: 10.1371/journal.pone.0361189 (PMC13712590; doi:10.1371/journal.pone.0361189)
Supplement: Peer review history

# Peer review: Decision Letter 0

**DOI:** 10.1371/journal.pone.0361189.r001

**Authors:** SSS Sarma

10 Sep 2026

Dear Dr. Buschini,

Thank you for submitting your manuscript to PLOS One. After careful consideration, we feel that it has merit but does not fully meet PLOS One’s publication criteria as it currently stands. Therefore, we invite you to submit a revised version of the manuscript that addresses the points raised during the review process.

Please submit your revised manuscript by Oct 10 2026 11:59PM. If you will need more time than this to complete your revisions, please reply to this message or contact the journal office at plosone@plos.org. When you're ready to submit your revision, log on to https://www.editorialmanager.com/pone/ and select the 'Submissions Needing Revision' folder to locate your manuscript file.

- A letter that responds to each point raised by the academic editor and reviewer(s). You should upload this letter as a separate file labeled 'Response to Reviewers'.
- A marked-up copy of your manuscript that highlights changes made to the original version. You should upload this as a separate file labeled 'Revised Manuscript with Track Changes'.
- An unmarked version of your revised paper without tracked changes. You should upload this as a separate file labeled 'Manuscript'.

If you would like to make changes to your financial disclosure, please include your updated statement in your cover letter. Guidelines for resubmitting your figure files are available below the reviewer comments at the end of this letter.

If applicable, we recommend that you deposit your laboratory protocols in protocols.io to enhance the reproducibility of your results. Protocols.io assigns your protocol its own identifier (DOI) so that it can be cited independently in the future. For instructions see: https://journals.plos.org/plosone/s/submission-guidelines#loc-laboratory-protocols. Additionally, PLOS One offers an option for publishing peer-reviewed Lab Protocol articles, which describe protocols hosted on protocols.io. Read more information on sharing protocols at https://plos.org/protocols?utm_medium=editorial-email&utm_source=authorletters&utm_campaign=protocols.

As the corresponding author, your ORCID iD is verified in the submission system and will appear in the published article. PLOS supports the use of ORCID, and we encourage all coauthors to register for an ORCID iD and use it as well. Please encourage your coauthors to verify their ORCID iD within the submission system before final acceptance, as unverified ORCID iDs will not appear in the published article. *Only*  the individual author can complete the verification step; PLOS staff *cannot*  verify ORCID iDs on behalf of authors.

We look forward to receiving your revised manuscript.

Kind regards,

SSS Sarma

Academic Editor

PLOS One

**Journal Requirements:**

When submitting your revision, we need you to address these additional requirements.

1. Please ensure that your manuscript meets PLOS ONE's style requirements, including those for file naming. The PLOS ONE style templates can be found at

https://journals.plos.org/plosone/s/file?id=wjVg/PLOSOne_formatting_sample_main_body.pdf and

https://journals.plos.org/plosone/s/file?id=ba62/PLOSOne_formatting_sample_title_authors_affiliations.pdf

2. Please note that PLOS One has specific guidelines on code sharing for submissions in which author-generated code underpins the findings in the manuscript. In these cases, we expect all author-generated code to be made available without restrictions upon publication of the work. Please review our guidelines at https://journals.plos.org/plosone/s/materials-and-software-sharing#loc-sharing-code and ensure that your code is shared in a way that follows best practice and facilitates reproducibility and reuse.

3. Thank you for stating the following in the Acknowledgments Section of your manuscript:

“This work has benefited from the equipment and framework of the COMP-R Initiatives, funded by the ‘Departments of Excellence’ program of the Italian Ministry for University and Research (MUR, 2023-2027). This work was also supported by the National Science Foundation (NSF) EPSCoR RII Track-2 FEC (OIA 1736026) to Riccardo Papa. The authors would like to thank Dr. Mirca Lazzaretti for her support with the graphic design.”

We note that you have provided funding information that is not currently declared in your Funding Statement. However, funding information should not appear in the Acknowledgments section or other areas of your manuscript. We will only publish funding information present in the Funding Statement section of the online submission form.

Please remove any funding-related text from the manuscript and let us know how you would like to update your Funding Statement. Currently, your Funding Statement reads as follows:

“The author(s) received no specific funding for this work.”

Please include your amended statements within your cover letter; we will change the online submission form on your behalf.

4. In the online submission form you indicate that your data is not available for proprietary reasons and have provided a contact point for accessing this data. Please note that your current contact point is a co-author on this manuscript. According to our Data Policy, the contact point must not be an author on the manuscript and must be an institutional contact, ideally not an individual. Please revise your data statement to a non-author institutional point of contact, such as a data access or ethics committee, and send this to us via return email. Please also include contact information for the third party organization, and please include the full citation of where the data can be found.

5. We note that Supporting Figure 4 in your submission may contain copyrighted images. All PLOS content is published under the Creative Commons Attribution License (CC BY 4.0), which means that the manuscript, images, and Supporting Information files will be freely available online, and any third party is permitted to access, download, copy, distribute, and use these materials in any way, even commercially, with proper attribution. For more information, see our copyright guidelines: http://journals.plos.org/plosone/s/licenses-and-copyright.

We require you to either (1) present written permission from the copyright holder to publish these figures specifically under the CC BY 4.0 license, or (2) remove the figures from your submission:

1. You may seek permission from the original copyright holder of Supporting Figure 4 to publish the content specifically under the CC BY 4.0 license.

We recommend that you contact the original copyright holder with the Content Permission Form (http://journals.plos.org/plosone/s/file?id=7c09/content-permission-form.pdf) and the following text:

“I request permission for the open-access journal PLOS ONE to publish XXX under the Creative Commons Attribution License (CCAL) CC BY 4.0 (http://creativecommons.org/licenses/by/4.0/). Please be aware that this license allows unrestricted use and distribution, even commercially, by third parties. Please reply and provide explicit written permission to publish XXX under a CC BY license and complete the attached form.”

Please upload the completed Content Permission Form or other proof of granted permissions as an "Other" file with your submission.

In the figure caption of the copyrighted figure, please include the following text: “Reprinted from [ref] under a CC BY license, with permission from [name of publisher], original copyright [original copyright year].”

2. If you are unable to obtain permission from the original copyright holder to publish these figures under the CC BY 4.0 license or if the copyright holder’s requirements are incompatible with the CC BY 4.0 license, please either i) remove the figure or ii) supply a replacement figure that complies with the CC BY 4.0 license. Please check copyright information on all replacement figures and update the figure caption with source information. If applicable, please specify in the figure caption text when a figure is similar but not identical to the original image and is therefore for illustrative purposes only.

6. If the reviewer comments include a recommendation to cite specific previously published works, please review and evaluate these publications to determine whether they are relevant and should be cited. There is no requirement to cite these works unless the editor has indicated otherwise.

7. Please review your reference list to ensure that it is complete and correct. If you have cited papers that have been retracted, please include the rationale for doing so in the manuscript text, or remove these references and replace them with relevant current references. Any changes to the reference list should be mentioned in the rebuttal letter that accompanies your revised manuscript. If you need to cite a retracted article, indicate the article’s retracted status in the References list and also include a citation and full reference for the retraction notice.

[Note: HTML markup is below. Please do not edit.]

Reviewers' comments:

Reviewer's Responses to Questions

**Comments to the Author**

1. Is the manuscript technically sound, and do the data support the conclusions?

Reviewer #1: Yes

Reviewer #2: Yes

**********

2. Has the statistical analysis been performed appropriately and rigorously?

Reviewer #1: Yes

Reviewer #2: Yes

**********

3. Have the authors made all data underlying the findings in their manuscript fully available?

The PLOS Data policy

Reviewer #1: Yes

Reviewer #2: Yes

**********

4. Is the manuscript presented in an intelligible fashion and written in standard English?

Reviewer #1: Yes

Reviewer #2: Yes

**********

Reviewer #1: Environmental modulation of methyl farnesoate release in two clones of Daphnia pulex differing in the sensitivity to exogenous stimuli

In general, the subject of the research is of interest and innovative, due to the exposition of the xenobiotics and the difference using two clones of Daphnia regarding the relation with the sex determination. This is far interesting due to the study addresses whether endogenous MF release changes under environmental conditions associated with sexual reproduction and phenotypic plasticity.

Abstract

Authors explain the trends in general over the exposition of MF however, no quantitative data is shown.

Introduction

While the data is of relevance, it is suggested that further explanation to why it remains unresolved. Line 73-74.

The examples of different studies are of relevance however, authors should consider adding some quantitative data or ranges to increase importance.

Additionally, when mentioning MF “production” can be modified for “released or accumulation in rearing medium…

In the section of discussion be careful of the terms, as authors manifest a mechanistic hypothesis when responding to crowding when reduced the MF in the medium.

During the experiments and interpretations density and food availability are considered in the same parameter, therefore no experimental was done. Can these parameters interfere? explain …

In the ms, body size is mentioned, however no follow-up was added.

Also, a graphical abstract would elevate the interest of the ms.

In general, the ms is of great scientific value, as no response to the question has been RESOLVED. However, these are great findings to give explanation.

Reviewer #2: Brief comments are provided below:

Line 150 – Item 2 is missing.

Line 194 – shows

Line 382 – Control

Lines 401–402 – Water samples were frozen at -20°C. Please comment on whether this is due to the compound's volatility or simply for storage purposes.

Line 413 – Control

**********

what does this mean?). If published, this will include your full peer review and any attached files.

If you choose “no”, your identity will remain anonymous but your review may still be made public.

**Do you want your identity to be public for this peer review?**  For information about this choice, including consent withdrawal, please see our Privacy Policy

Reviewer #1: **Yes:**  Brenda Karen González Pérez

Reviewer #2: No

**********

[NOTE: If reviewer comments were submitted as an attachment file, they will be attached to this email and accessible via the submission site. Please log into your account, locate the manuscript record, and check for the action link "View Attachments". If this link does not appear, there are no attachment files.]

To ensure your figures meet our technical requirements, please review our figure guidelines: https://journals.plos.org/plosone/s/figures

You may also use PLOS’s free figure tool, NAAS, to help you prepare publication quality figures: https://journals.plos.org/plosone/s/figures#loc-tools-for-figure-preparation.

NAAS will assess whether your figures meet our technical requirements by comparing each figure against our figure specifications.

# Author comment: Author response to Decision Letter 1

**DOI:** 10.1371/journal.pone.0361189.r002

2 Oct 2026

PONE-D-26-38849

Environmental modulation of methyl farnesoate release in two clones of Daphnia pulex differing in the sensitivity to exogenous stimuli

Editor’s Comments

Thank you for submitting your manuscript to PLOS One. After careful consideration, we feel that it has merit but does not fully meet PLOS One’s publication criteria as it currently stands. Therefore, we invite you to submit a revised version of the manuscript that addresses the points raised during the review process.

Journal Requirements:

When submitting your revision, we need you to address these additional requirements.

1. Please ensure that your manuscript meets PLOS ONE's style requirements, including those for file naming. The PLOS ONE style templates can be found at

https://journals.plos.org/plosone/s/file?id=wjVg/PLOSOne_formatting_sample_main_body.pdf and

https://journals.plos.org/plosone/s/file?id=ba62/PLOSOne_formatting_sample_title_authors_affiliations.pdf

We thank the Editor for this suggestion. The revised version of the manuscript was prepared following PLOS ONE's style requirements.

2. Please note that PLOS One has specific guidelines on code sharing for submissions in which author-generated code underpins the findings in the manuscript. In these cases, we expect all author-generated code to be made available without restrictions upon publication of the work. Please review our guidelines at https://journals.plos.org/plosone/s/materials-and-software-sharing#loc-sharing-code and ensure that your code is shared in a way that follows best practice and facilitates reproducibility and reuse.

The authors did not generate any code.

3. Thank you for stating the following in the Acknowledgments Section of your manuscript:

“This work has benefited from the equipment and framework of the COMP-R Initiatives, funded by the ‘Departments of Excellence’ program of the Italian Ministry for University and Research (MUR, 2023-2027). This work was also supported by the National Science Foundation (NSF) EPSCoR RII Track-2 FEC (OIA 1736026) to Riccardo Papa. The authors would like to thank Dr. Mirca Lazzaretti for her support with the graphic design.”

We note that you have provided funding information that is not currently declared in your Funding Statement. However, funding information should not appear in the Acknowledgments section or other areas of your manuscript. We will only publish funding information present in the Funding Statement section of the online submission form.

Please remove any funding-related text from the manuscript and let us know how you would like to update your Funding Statement. Currently, your Funding Statement reads as follows:

“The author(s) received no specific funding for this work.”

Please include your amended statements within your cover letter; we will change the online submission form on your behalf.

We thank the Editor for this comment: the Acknowledgments Section was modified accordingly.

4. In the online submission form you indicate that your data is not available for proprietary reasons and have provided a contact point for accessing this data. Please note that your current contact point is a co-author on this manuscript. According to our Data Policy, the contact point must not be an author on the manuscript and must be an institutional contact, ideally not an individual. Please revise your data statement to a non-author institutional point of contact, such as a data access or ethics committee, and send this to us via return email. Please also include contact information for the third party organization, and please include the full citation of where the data can be found.

The institutional reference address for data access is: monitoraggioricerca@unipr.

Furthermore, we have deposited the data and R scripts used for the statistical analyses in Zenodo (https://doi.org/10.5281/zenodo.22935162 - link: https://zenodo.org/records/22935162) and added the DOI to the Data Availability Statement and to the Statistical analysis section of the revised manuscript.

5. We note that Supporting Figure 4 in your submission may contain copyrighted images. All PLOS content is published under the Creative Commons Attribution License (CC BY 4.0), which means that the manuscript, images, and Supporting Information files will be freely available online, and any third party is permitted to access, download, copy, distribute, and use these materials in any way, even commercially, with proper attribution. For more information, see our copyright guidelines: http://journals.plos.org/plosone/s/licenses-and-copyright.

We require you to either (1) present written permission from the copyright holder to publish these figures specifically under the CC BY 4.0 license, or (2) remove the figures from your submission:

1. You may seek permission from the original copyright holder of Supporting Figure 4 to publish the content specifically under the CC BY 4.0 license.

We recommend that you contact the original copyright holder with the Content Permission Form (http://journals.plos.org/plosone/s/file?id=7c09/content-permission-form.pdf) and the following text:

“I request permission for the open-access journal PLOS ONE to publish XXX under the Creative Commons Attribution License (CCAL) CC BY 4.0 (http://creativecommons.org/licenses/by/4.0/). Please be aware that this license allows unrestricted use and distribution, even commercially, by third parties. Please reply and provide explicit written permission to publish XXX under a CC BY license and complete the attached form.”

Please upload the completed Content Permission Form or other proof of granted permissions as an "Other" file with your submission.

In the figure caption of the copyrighted figure, please include the following text: “Reprinted from [ref] under a CC BY license, with permission from [name of publisher], original copyright [original copyright year].”

2. If you are unable to obtain permission from the original copyright holder to publish these figures under the CC BY 4.0 license or if the copyright holder’s requirements are incompatible with the CC BY 4.0 license, please either i) remove the figure or ii) supply a replacement figure that complies with the CC BY 4.0 license. Please check copyright information on all replacement figures and update the figure caption with source information. If applicable, please specify in the figure caption text when a figure is similar but not identical to the original image and is therefore for illustrative purposes only.

Thank you for bringing this matter to our attention. We confirm that Supporting Figure 4, entitled “S4 Fig. Distribution of morphotypes in clones I and S across population density and photoperiod conditions,” does not contain any third-party copyrighted material. All photographs show specimens obtained during the present study and were taken by the authors. The photographs have not been previously published, and the authors own the copyright and agree to their publication under the Creative Commons Attribution License (CC BY 4.0). Therefore, permission from an external copyright holder and submission of a Content Permission Form are not required.

6. If the reviewer comments include a recommendation to cite specific previously published works, please review and evaluate these publications to determine whether they are relevant and should be cited. There is no requirement to cite these works unless the editor has indicated otherwise.

We carefully reviewed the reviewers’ comments for recommendations to cite specific previously published works. The reviewers did not recommend the citation of any specific publication. Therefore, no references were added in response to specific comments of the reviewers.

7. Please review your reference list to ensure that it is complete and correct. If you have cited papers that have been retracted, please include the rationale for doing so in the manuscript text, or remove these references and replace them with relevant current references. Any changes to the reference list should be mentioned in the rebuttal letter that accompanies your revised manuscript. If you need to cite a retracted article, indicate the article’s retracted status in the References list and also include a citation and full reference for the retraction notice.

Thank you for this comment. We carefully reviewed the complete reference list for accuracy, completeness, and publication status. No cited articles were found to have been retracted, and therefore no retracted publications or associated retraction notices are included in the manuscript. We corrected the publication year and added the DOI for reference 13, completed the DOI reported in reference 31, and added the missing DOI to reference 36. We also added the reference by Teal et al. (2014) to support the statement concerning the identification of methyl farnesoate by two-dimensional nuclear magnetic resonance spectroscopy.

Reviewers' comments

Reviewer #1: Environmental modulation of methyl farnesoate release in two clones of Daphnia pulex differing in the sensitivity to exogenous stimuli. In general, the subject of the research is of interest and innovative, due to the exposition of the xenobiotics and the difference using two clones of Daphnia regarding the relation with the sex determination. This is far interesting due to the study addresses whether endogenous MF release changes under environmental conditions associated with sexual reproduction and phenotypic plasticity.

Abstract

Authors explain the trends in general over the exposition of MF however, no quantitative data is shown.

We thank the Reviewer for the comment, the abstract was modified accordingly by adding quantitative data related to MF release (lines 34-41).

Introduction

While the data is of relevance, it is suggested that further explanation to why it remains unresolved. Line 72-74.

We thank the reviewer for this comment. We have expanded this section to clarify why the endogenous role of MF in Daphnia remains unresolved by adding the following sentence: “However, available evidence mainly derives from phenotypic responses to exogenous MF, pharmacological manipulation of the putative signaling pathway, or changes in the expression of genes involved in MF/JH biosynthesis. MF has not yet been chemically characterized within Daphnia tissues or hemolymph. The detection of MF in Daphnia culture water provides indirect evidence of its release but does not establish its synthesis within the organism or demonstrate that environmentally induced variations in endogenous MF directly cause reproductive or defensive phenotypes.”(lines 77-83)

The examples of different studies are of relevance however, authors should consider adding some quantitative data or ranges to increase importance.

We thank the reviewer for this suggestion. The Introduction has been revised to include representative quantitative information from previous studies: “For example, exposure to juvenile hormone III induced male production with an EC50 of approximately 4 µM, whereas MF concentrations of 48–50 µg/L produced an approximately 1:1 offspring sex ratio in D. magna [7]. Methoprene concentrations up to 160 nM did not induce male production under female-promoting conditions but produced a concentration-dependent response when combined with crowding and food restriction [7]. These findings demonstrate that JH-like compounds can alter sex determination; however, the concentrations used in exogenous exposure experiments are substantially higher than the endogenous MF levels expected in the surrounding aquatic medium.” Lines 65-72. In addition, the concentration of MF detected in water medium was added in line 114.

Additionally, when mentioning MF “production” can be modified for “released or accumulation in rearing medium…

We thank the reviewer for this comment. We agree that our measurements quantify MF released into and accumulated in the rearing medium rather than its biosynthetic production within the organism. We therefore reviewed the manuscript and revised potentially ambiguous occurrences of the term “production”. In the Materials and Methods section, the statement “assuming the MF was produced by adult females over 48 h” has been replaced with “allowing MF released by adult females to accumulate in the rearing medium over 48 h” (lines 194-195). We also revised the corresponding statement in the Conclusions to refer to the environmental factors controlling MF release and accumulation in the rearing medium. The term “production” has been retained only where it explicitly refers to the biological hypothesis of endogenous MF synthesis or to the production of males, ephippia, or offspring.

In the section of discussion be careful of the terms, as authors manifest a mechanistic hypothesis when responding to crowding when reduced the MF in the medium.

We thank the reviewer for this important observation. We agree that the decrease in MF concentration in the rearing medium under crowding should not be interpreted as evidence of a specific mechanistic response or of reduced endogenous MF production. We have therefore revised the Discussion to distinguish the measured concentration in the medium from MF biosynthesis within the organism (lines 449-456).

During the experiments and interpretations density and food availability are considered in the same parameter, therefore no experimental was done. Can these parameters interfere? explain …

We thank the reviewer for raising this important point. We agree that population density and food availability per individual may interact and that their effects cannot be separated in the second experiment. In the first experiment, crowding was generated by reducing the available water volume while maintaining the number of daphnids and the food ration per individual; this design therefore evaluated the effect of reduced available space without introducing food limitation. In the second experiment, by contrast, population density was increased while food concentration was kept constant. Consequently, food availability per individual decreased as density increased, and the observed responses represent the combined effects of increasing population density, greater interaction among individuals and decreasing food availability per individual.

In a previous paper we described the separate effect of crowding (HFHD, High food (1.5 × 105 cells/ml of P. subcapitata and of S. cerevisiae) and high daphnid density (1 individual/10 ml)) and food shortage due to low algae and yeast cell density (LFLD treatment Low food (0.3 × 105 cells/ml of P. subcapitata and of S. cerevisiae) and low daphnid density (1individual/50 ml)) in clone I and clone S (Suppa et al. 2021b). In both clones, I and S, the decrease of fecundity was significantly more pronounced under crowded conditions (HFHD treatment) than in conditions of food shortage due to low algae and yeast cell density (LFLD treatment). No male offspring and ephippia were observed in the progeny of clone I in any of the treatments (HFHD, LFLD and HFLD High food (1.5 × 105 cells/ml of P. subcapitata and of Saccharomyces cerevisiae) and low daphnid density (1 individual/50 ml)). Clone S produced males in all three treatments, with the highest percentage in the HFHD treatment, and produced ephippia only in the crowded condition (HFHD). These previous results suggest that density-related crowding has a stronger effect than food limitation alone. We have therefore revised the manuscript to state explicitly that the present experimental design does not allow their individual contributions or interaction to be quantified, and we interpret the results as the response to a combined density-food availability treatment (lines 389-396).

In the ms, body size is mentioned, however no follow-up was added

We thank the reviewer for this comment. Body size was assessed as mean carapace length and the corresponding results are presented in Fig 4A. To provide a clearer follow-up, we have revised the Results (lines 316-318) and expanded the Discussion (lines 402-406). The revised text clarifies that females of both clones were smaller under the conditions simulating early spring than under those simulating late spring. In clone S, the difference between the two seasonal conditions decreased at the highest population densities, whereas no statistically significant effect of density on body size was detected in clone I. The Discussion now explicitly considers these clone-specific responses in relation to sensitivity to population density and seasonal conditions.

Also, a graphical abstract would elevate the interest of the ms.

We thank the Reviewer for this suggestion: the graphical abstract was provided along with the revised version of the manuscript.

In general, the ms is of great scientific value, as no response to the question has been RESOLVED. However, these are great findings to give explanation.

We thank the reviewer for this valuable comment, we are seeking to gain a deeper understanding of the role of MF; although we hypothesize that it may be dual (hormone/pheromone), the research still requires further investigation.

Reviewer #2: Brief comments are provided below:

Line 150 – Item 2 is missing.

We thank the referee for checking the manuscript: as requested the numbering was added

Line 194 – shows

Thank you again: as requested by the Referee, the typo was corrected

Line 382 – Control

Regarding this comment, we would like to point out that we used the abbreviation “Contr” consistently throughout the manuscript. The abbreviations are defined in lines 170-178. Therefore, we prefer to retain the term “Contr” rather than replace it with “Control” to ensure consistency throughout the manuscript.

Lines 401–402 – Water samples were frozen at -20°C. Please comment on whether this is due to the compound's volatility or simply for storage purposes.

We thank the reviewer for this comment. Water samples were stored at −20°C to preserve MF stability during storage. Freezing was intended to minimize potential analyte loss due to evaporation, as well as to limit potential chemical or enzyme-mediated degradation before analysis. A new sentence was added to the revised manuscript line 179-180.

Line 413 – Control

Please, see our previous response.

Attachment Submitted filename: response to reviewers.docx

# Editor report: Decision Letter 1

**DOI:** 10.1371/journal.pone.0361189.r003

**Authors:** SSS Sarma

8 Oct 2026

Environmental modulation of methyl farnesoate release in two clones of Daphnia pulex differing in the sensitivity to exogenous stimuli

PONE-D-26-38849R1

Dear Dr. Buschini,

We’re pleased to inform you that your manuscript has been judged scientifically suitable for publication and will be formally accepted for publication once it meets all outstanding technical requirements.

Within one week, you’ll receive an e-mail detailing the required amendments. When these have been addressed, you’ll receive a formal acceptance letter and your manuscript will be scheduled for publication.

An invoice will be generated when your article is formally accepted. Please note, if your institution has a publishing partnership with PLOS and your article meets the relevant criteria, all or part of your publication costs will be covered. Please make sure your user information is up-to-date by logging into Editorial Manager at Editorial Manager® and clicking the ‘Update My Information' link at the top of the page. For questions related to billing, please contact billing support.

If your institution or institutions have a press office, please notify them about your upcoming paper to help maximize its impact. If they’ll be preparing press materials, please inform our press team as soon as possible -- no later than 48 hours after receiving the formal acceptance. Your manuscript will remain under strict press embargo until 2 pm Eastern Time on the date of publication. For more information, please contact onepress@plos.org.

Kind regards,

SSS Sarma

Academic Editor

PLOS One

Additional Editor Comments (optional):

Reviewers' comments:

# Editor report: Acceptance letter

**DOI:** 10.1371/journal.pone.0361189.r004

**Authors:** SSS Sarma

PONE-D-26-38849R1

PLOS One

Dear Dr. Buschini,

I'm pleased to inform you that your manuscript has been deemed suitable for publication in PLOS One. Congratulations! Your manuscript is now being handed over to our production team.

At this stage, our production department will prepare your paper for publication. This includes ensuring the following:

* All references, tables, and figures are properly cited

* All relevant supporting information is included in the manuscript submission,

* There are no issues that prevent the paper from being properly typeset

You will receive further instructions from the production team, including instructions on how to review your proof when it is ready. Please keep in mind that we are working through a large volume of accepted articles, so please give us a few days to review your paper and let you know the next and final steps.

Lastly, if your institution or institutions have a press office, please let them know about your upcoming paper now to help maximize its impact. If they'll be preparing press materials, please inform our press team within the next 48 hours. Your manuscript will remain under strict press embargo until 2 pm Eastern Time on the date of publication. For more information, please contact onepress@plos.org.

You will receive an invoice from PLOS for your publication fee after your manuscript has reached the completed accept phase. If you receive an email requesting payment before acceptance or for any other service, this may be a phishing scheme. Learn how to identify phishing emails and protect your accounts at https://explore.plos.org/phishing.

If we can help with anything else, please email us at customercare@plos.org.

Thank you for submitting your work to PLOS One and supporting open access.

Kind regards,

PLOS One Editorial Office Staff

on behalf of

Professor SSS Sarma

Academic Editor

PLOS One
